# The ciliary gene *INPP5E* confers dorsal telencephalic identity to human cortical organoids by negatively regulating Sonic hedgehog signaling

**DOI:** 10.1016/j.celrep.2022.110811

**Published:** 2022-05-17

**Authors:** Leah Schembs, Ariane Willems, Kerstin Hasenpusch-Theil, James D. Cooper, Katie Whiting, Karen Burr, Sunniva M.K. Bøstrand, Bhuvaneish T. Selvaraj, Siddharthan Chandran, Thomas Theil

**Affiliations:** 1 Centre for Discovery Brain Sciences, Hugh Robson Building, University of Edinburgh, Edinburgh EH8 9XD, UK; 2 Centre for Clinical Brain Sciences, University of Edinburgh, Edinburgh EH16 4SB, UK; 3 UK Dementia Research Institute at University of Edinburgh, University of Edinburgh, Edinburgh EH16 4SB, UK; 4 Simons Initiative for the Developing Brain, University of Edinburgh, Hugh Robson Building, Edinburgh EH8 9XD, UK; 5 Anne Rowling Regenerative Neurology Clinic, University of Edinburgh, Edinburgh EH16 4SB, UK

**Keywords:** *INPP5E*, primary cilia, sonic hedgehog, dorsal and ventral patterning, human cortex, telencephalon

## Abstract

Defects in primary cilia, cellular antennas that control multiple intracellular signaling pathways, underlie several neurodevelopmental disorders, but it remains unknown how cilia control essential steps in human brain formation. Here, we show that cilia are present on the apical surface of radial glial cells in human fetal forebrain. Interfering with cilia signaling in human organoids by mutating the *INPP5E* gene leads to the formation of ventral telencephalic cell types instead of cortical progenitors and neurons. *INPP5E* mutant organoids also show increased Sonic hedgehog (SHH) signaling, and cyclopamine treatment partially rescues this ventralization. In addition, ciliary expression of SMO, GLI2, GPR161, and several intraflagellar transport (IFT) proteins is increased. Overall, these findings establish the importance of primary cilia for dorsal and ventral patterning in human corticogenesis, indicate a tissue-specific role of *INPP5E* as a negative regulator of SHH signaling, and have implications for the emerging roles of cilia in the pathogenesis of neurodevelopmental disorders.

## Highlights

• A mutation in the ciliary gene *INPP5E* leads to ventralization of cortical organoids
• *INPP5E* mutant organoids show an up-regulation of SHH signaling
• Inhibiting SHH signaling partially rescues the ventralization phenotype
• *INPP5E* mutant primary cilia accumulate SMO, GPR161, and several IFT proteins

## Graphical abstract

**Figure:**
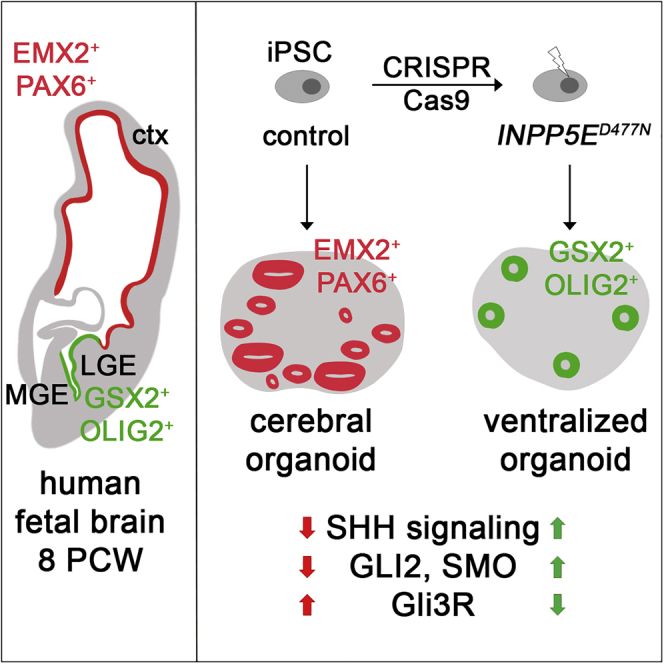

## Teaser

Schembs et al. investigate the role of primary cilia in human corticogenesis using *INPP5E* mutant cerebral organoids. The analyses show that overactive SHH signaling causes the formation of ventral telencephalic progenitors and neurons instead of cortical cells and imply important ciliary functions in the pathogenesis of neurodevelopmental disorders.

## Introduction

The cerebral cortex is the most complex part of the human brain and confers humans with their unique cognitive capabilities. The cellular and molecular mechanisms governing corticogenesis are under intensive investigation since cortical malformations underlie a number of neurodevelopmental disorders, such as autism spectrum disorders (ASDs) and intellectual disability (ID). The cortex develops from the most rostral part of the brain, the telencephalon. A patterning process subdivides the telencephalon into distinct dorsal and ventral territories, forming the cortex and the basal ganglia, respectively. The molecular basis for this process is well understood in mice (Rallu et al., 2002a), where dorsal progenitor cells express the Pax6 and Emx transcription factors and generate glutamatergic projection neurons either directly or indirectly via Tbr2^+^ basal progenitors (Englund et al., 2005; Götz et al., 1998; Warren et al., 1999). The ventral telencephalon is further subdivided into the caudal, medial, and lateral ganglionic eminences (CGE, MGE, and LGE, respectively) that express the Nkx2.1, Gsx2, Dlx2, and Olig2 transcription factors and produce a variety of different neuronal subtypes and oligodendrocyte precursor cells (OPCs). Notably, GABAergic cortical interneurons are formed in the MGE and CGE from where they migrate tangentially into the cortex (Marin and Muller, 2014). Generating these subdivisions of the telencephalon with their characteristic combinatorial expression of transcription factors is under the control of several signaling molecules (Theil et al., 2002). Members of the Wnt and Bmp gene families produced in the cortical hem promote cortical development, whereas Shh signaling induces the formation of ventral telencephalic structures and cell types (Rallu et al., 2002a). Thus, a combination of signaling molecules and transcription factors direct murine corticogenesis; however, due to species-specific cellular features of the human brain (Florio and Huttner, 2014; Hodge et al., 2019) and due to a lack of suitable human model systems, it remains largely unknown whether this mechanism is evolutionarily conserved between mice and humans. In particular, it has not been explored how telencephalic signaling is regulated at the cellular level during human corticogenesis and how defects in cell signaling could contribute to neurodevelopmental disorders.

Primary cilia are small, microtubule-based protrusions from the cell surface that act as antennae in the detection and intracellular transduction of multiple cell-cell signals. Defects in the function and/or structure of primary cilia underlie a group of syndromes referred to as ciliopathies (Tobin and Beales, 2009). Ciliopathies are characterized by pleiotropic clinical features, and many ciliopathy patients display severe neurological symptoms, most commonly ID (Valente et al., 2014). In turn, many candidate genes for ASD, schizophrenia, and ID affect primary cilia function (Canning et al., 2018; Guo et al., 2017; Lee and Gleeson, 2011; Louvi and Grove, 2011; Marley and von Zastrow, 2012). Despite the emerging role of primary cilia in neurodevelopmental disorders, their participation in the underlying disease mechanisms remains largely unexplored. In particular, a better understanding of their involvement in physiological human brain development is required.

Sonic hedgehog (SHH) signaling is a key factor in normal and abnormal nervous system development. Crucially, its pathway activity is controlled by primary cilia. In the absence of SHH protein, ciliary expression levels of SMO, the main cellular transducer of hedgehog signals, are low, since PTCH1 prevents SMO from entering the cilium (Rohatgi et al., 2007). Activation of protein kinase A (PKA) through the negative regulator GPR161 (Mukhopadhyay et al., 2013) results in the phosphorylation of the GLI3 transcription factor, its proteolytic processing at the ciliary base, and the formation of the GLI3 repressor (GLI3R) (Haycraft et al., 2005). In contrast, upon pathway activation, SHH binds to and represses PTCH1 so that SMO together with SUFU, GLI2, and GLI3 accumulate at the ciliary tip (Corbit et al., 2005; Haycraft et al., 2005; Kopinke et al., 2020; Liem et al., 2009; Rohatgi et al., 2007; Wen et al., 2010). There, the GLI proteins are converted through phosphorylation events into transcriptional activators (GLIA) (Han et al., 2019) that translocate to the nucleus and stimulate the transcription of SHH target genes. Thus, primary cilia are critical for both producing GLI3R and for activating the full-length GLI proteins. These activities make cilia ideal candidates for controlling human nervous system development, a function that has hitherto not been examined in detail in human model systems.

Due to their involvement in cell-cell signaling in general and in SHH signaling in particular, primary cilia are likely to be crucial in controlling dorsal and ventral (D/V) patterning of the telencephalon (Andreu-Cervera et al., 2021; Liu et al., 2021; Park et al., 2019). Indeed, several ciliary mouse mutants show a variety of telencephalic patterning phenotypes ranging from extensive ventralization of the dorsal telencephalon to milder defects in forming the pallial/subpallial boundary that separates the dorsal from the ventral telencephalon (Ashique et al., 2009; Besse et al., 2011; Hasenpusch-Theil et al., 2020; Stottmann et al., 2009; Willaredt et al., 2008). To address whether cilia play a similar role in human corticogenesis, we became interested in the ciliary gene *INPP5E* that is mutated in mental retardation, obesity, retinal dystrophy, and micropenis (MORM) syndrome (Jacoby et al., 2009) and in Joubert syndrome (JS) (Bielas et al., 2009), a ciliopathy characterized by cerebellar defects and malformations of the cerebral cortex in a subset of patients. *INPP5E* encodes inositol polyphosphate-5-phosphatase E, a ciliary-membrane-associated protein that hydrolyzes the phosphatidylinositol polyphosphates PI(4,5)P_2_ and PI(3,4,5)P_3_ (Bielas et al., 2009; Jacoby et al., 2009). In this way, INPP5E creates a specific phosphoinositol composition of the ciliary membrane that has been linked to protein trafficking to either repress or activate hedgehog signaling in a tissue-specific manner (Chavez et al., 2015; Constable et al., 2020; Garcia-Gonzalo et al., 2015; Hasenpusch-Theil et al., 2020). *Inpp5e* also regulates cilia assembly (Xu et al., 2016), stability (Jacoby et al., 2009), and disassembly (Phua et al., 2017; Plotnikova et al., 2015). Moreover, *Inpp5e* knockout and conditional mouse mutants recapitulate many of the defects observed in human JS patients (Hakim et al., 2016; Hasenpusch-Theil et al., 2020; Jacoby et al., 2009; Ukhanov et al., 2021). In particular, we recently showed that loss of *Inpp5e* leads to a defective pallial/subpallial boundary and has profound effects on cortical stem cell functions (Hasenpusch-Theil et al., 2020).

Based on these critical roles in ciliary biology and in mammalian development, *INPP5E* presents an excellent candidate to study potential functions of primary cilia in human corticogenesis. Using CRISPR-Cas9 mutagenesis, we engineered human induced pluripotent stem cells (iPSCs) to carry an *INPP5E* loss-of-function mutation and generated cortical organoids from these lines. We show that inactivating *INPP5E* resulted in ventralized organoids that formed ventral telencephalic progenitors and neurons rather than cortical projection neurons. The mutation also caused an up-regulation of SHH signaling that was necessary and sufficient for this ventralization. Mechanistically, we show that the *INPP5E* mutation led to an increased accumulation of SMO, GLI2, and several intraflagellar transport (IFT) proteins in the cilium.

## Results

### Primary cilia in the developing human telencephalon

**Figure 1:**
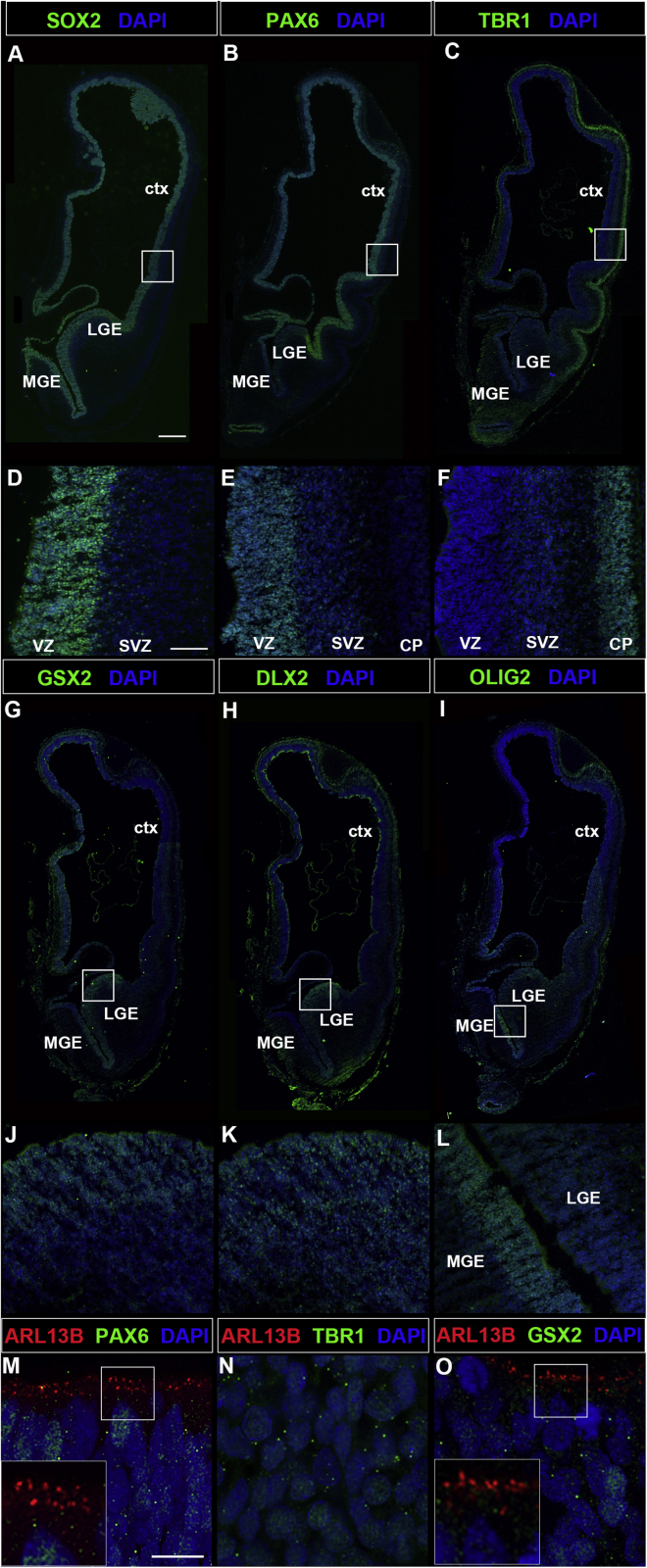
Cilia in the developing human telencephalon Coronal sections of the 8 PCW telencephalon immunostained with the indicated markers. (A and D) SOX2 expression in dorsal and ventral telencephalic progenitors. (B, C, E, and F) PAX6 and TBR1 expression are confined to dorsal progenitors and neurons, respectively. (G–L) GSX2 (G and J), DLX2 (H and K), and OLIG2 (I and L) expression are confined to the ventral telencephalon. (M–O) ARL13B^+^ cilia projecting from progenitor cells into the ventricular lumen in the dorsal (M) and ventral (O) telencephalon. Note the absence of ARL13B expression in TBR1^+^ projection neurons (N). ctx, cortex; LGE, lateral ganglionic eminence; MGE, medial ganglionic eminence; SVZ, subventricular zone; VZ, ventricular zone. Scale bars, 500 μm (A), 100 μm (D), and 2.5 μm (M). See also Figure S1.

Cell-cell signaling mediated by primary cilia plays crucial roles in murine corticogenesis (Andreu-Cervera et al., 2021; Hasenpusch-Theil and Theil, 2021; Liu et al., 2021). As a first step to establish ciliary function in human cortical development, we investigated the presence of primary cilia in the developing human telencephalon and immunostained the forebrain of a postconceptional week (PCW) 8 human embryo to reveal its D/V subdivisions. At this stage, SOX2 was expressed in progenitor cells in the ventricular zone (VZ) throughout the telencephalon with some scattered SOX2^+^ progenitors in the forming cortical subventricular zone (SVZ) (Figures 1A and 1D). PAX6 expression was confined to the dorsal telencephalon, where the protein was detected in apical radial glial cells (aRGCs) in the VZ and in a few progenitors in the SVZ with a lateral high to medial low expression gradient (Figures 1B and 1E) as described previously (Clowry et al., 2018; Qin et al., 2020). Moreover, cortical projection neurons residing in the forming cortical plate were identified by the expression of TBR1 (Figures 1C and 1F). In contrast, VZ progenitor cells of the lateral ganglionic eminence expressed GSX2 and DLX2 that was also detected in the SVZ (Figures 1G, 1H, 1J, and 1K). Finally, OLIG2 is expressed in the MGE and LGE progenitor domains and in scattered OLIG2^+^ oligodendrocyte precursor cells (Figures 1I and 1L). Immunostainings for some of these regional markers in combination with the axonemal marker ARL13B revealed the presence of primary cilia projecting from the apical surface of radial glial cells into the lumen of the lateral ventricle in both the cortex and the LGE (Figures 1M and 1O). Cilia were, however, not found on cortical projection neurons (Figure 1N). Thus, cilia are present on progenitor cells to receive signals important for D/V patterning of the human telencephalon.

### Generation and initial characterization of *INPP5E* mutant iPSCs

To test the role of primary cilia in telencephalon development, we focused on the *INPP5E* gene that is essential for cilia-mediated signaling (Bielas et al., 2009; Chavez et al., 2015; Garcia-Gonzalo et al., 2015; Jacoby et al., 2009) in neural stem cells (Chavez et al., 2015; Hasenpusch-Theil et al., 2020). To this end, we generated mutant human iPSC lines with a homozygous D477N mutation (Figure S1) using a CRISPR-Cas9 approach. This mutation is an enzymatic null mutation and has been widely used to characterize *INPP5E* function (Kong et al., 2006). A guide RNA (gRNA) was selected that had at least three mismatches to potential off-target sites, highly homologous to the on-target site. The gRNA/Cas9 plasmid was co-transfected with a template oligonucleotide carrying the D477N mutation into control iPSCs (Johnstone et al., 2019; Selvaraj et al., 2018; Vasistha et al., 2019). We achieved an ∼8% mutation efficiency and identified 23 clones with the desired homozygous mutation as confirmed by restriction fragment length polymorphism and Sanger sequencing (Figure S1). Two of these clones (iPSM1 and iPSM2) were chosen for further analyses. The mutant clones were karyotypically normal and retained the pluripotency markers NANOG, OCT3/4, and TRA-1-60 (Figure S2; Data S1). In both clones, we did not detect off-target activity of the gRNA for the six highest candidate off-target sites as assessed by Sanger sequencing.

Three control and two *INPP5E* mutant iPSC lines were differentiated into cerebral organoids using a modified Lancaster protocol (Figure S1; Lancaster et al., 2013). After dual-Smad inhibition stimulating neural induction and embryoid body (EB) formation, FGF2 was added to the culture medium to promote neuroepithelial expansion. EBs were maintained on a shaking incubator to enhance oxygen exchange and nutrient absorption. At week 4, control and mutant organoids developed large neuroepithelial loops that increased over the next couple of weeks before cerebral organoids were harvested at day 39 (D39). We first characterized the presence of primary cilia in these organoids. Control organoids form large, elongated neuroepithelia, whereas mutant organoids often formed smaller, rosette-like structures. ARL13B and γTUB staining labeling the ciliary axoneme and the basal body, respectively, revealed primary cilia emanating from apical radial glia cells into the lumen in organoids of both genotypes as found in human fetal cortical tissue (Figure S1). Moreover, INPP5E protein was confined to the ciliary axoneme and excluded from the basal body in control and mutant organoids, though the expression of the INPP5E^D477N/D477N^ mutant protein was reduced (Figure S1). To gain insights into ciliary stability that might be affected by the *INPP5E* mutation (Hasenpusch-Theil et al., 2020), we stained for glutamylated TUBULIN but could not detect a change in the frequency of glutamylated TUB^+^ cilia or in the glutamylated TUB/ARL13B intensity ratio. This analysis, however, revealed a shortening of cilia (Figure S3). Taken together, these findings indicate that control and *INPP5E*^D477N/D477N^ mutant cortical organoids establish a correct organization of the neuroepithelium with respect to the apical location of primary cilia and confined INPP5E protein expression in the axoneme.

### *INPP5E*^D477N/D477N^ organoids are ventralized

**Figure 2:**
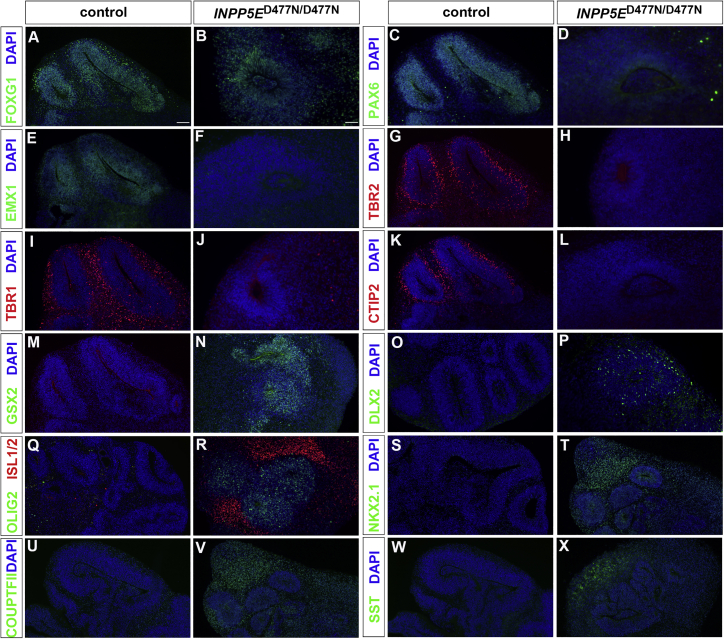
The *INPP5E* mutation interferes with telencephalic marker gene expression Control and *INPP5E*^D477N/D477N^ organoids were immunostained with the indicated markers. (A and B) The telencephalon marker FOXG1 was expressed in organoids of both genotypes. (C–F) The dorsal aRGC markers PAX6 (C and D) and EMX1 (E and F) were expressed in control, but not in mutant, organoids. (G and H) TBR2-labeled basal progenitor cells that were largely absent in *INPP5E*^D477N/D477N^ organoids. (I–L) Expression of the cortical neuron markers TBR1 and CTIP2 was largely absent in mutant organoids. (M–R) Expression of the ventral telencephalic progenitor markers GSX2 (M and N), DLX2 (O and P), and OLIG2 (Q and R) in mutant, but not in control, organoids. ISL1/2 labeling spiny medium interneurons was detected in *INPP5E* mutant organoids (Q and R). (S–X) Expression analyses of the ventral telencephalic interneuron markers NKX2.1 (S and T), COUPTFII (U and V), and SST (W and X). Scale bars, 100 μm (A) and 50 μm (B). See also Figure S3.

We next investigated the type of neuroepithelium formed in control and *INPP5E*^D477N/D477N^ mutant organoids. *FOXG1* encodes a transcription factor expressed throughout the telencephalon (Xuan et al., 1995) and was expressed in neural progenitors and neurons of both genotypes, indicating that the organoids acquired telencephalic identity (Figures 2A and 2B). To determine the regional telencephalic identity, we performed immunofluorescence analyses with various dorsal- and ventral-specific markers. PAX6 and EMX1 label aRGCs in the developing cortex, but these markers were hardly expressed in *INPP5E*^D477N/D477N^ organoids (Figures 2C–2F). During cortical neurogenesis, aRGCs undergo asymmetric cell divisions to form cortical neurons directly or indirectly via the production of basal progenitors. Basal progenitors and early-born cortical neurons are characterized by the expression of TBR2 and TBR1/CTIP2, respectively (Englund et al., 2005). TBR2^+^, TBR1^+^, and CTIP2^+^ cells were readily identified in control organoids separated into different layers in a similar arrangement to that found during corticogenesis (Figures 2G, 2I, and 2K). Interestingly, TBR2, TBR1, and CTIP2 expression was largely absent from mutant organoids (Figures 2H, 2J, and 2L), suggesting that the *INPP5E*^D477N/D477N^ mutation interferes with the formation of major dorsal telencephalic cell types.

The expression of FOXG1 and the lack of dorsal marker protein expression raise the possibility that *INPP5E* mutant organoids form ventral telencephalic progenitors and their neuronal derivatives. In embryonic development, the ventral telencephalon contains GSX2^+^ and DLX2^+^ progenitors and OLIG2^+^ oligodendrocyte precursor cells (OPCs). In mouse embryogenesis and in human organoids, OPCs are formed earlier in the ventral telencephalon compared with the cortex where OPC formation is delayed (Kessaris et al., 2006; Kim et al., 2019). In *INPP5E*^D477N/D477N^ mutant organoids, GSX2^+^, DLX2^+^, and OLIG2^+^ progenitors were found abundantly, whereas controls only contained a few isolated cells (Figures 2M–2R). We also determined the expression of markers characteristic of ventral telencephalic neurons. Striatal cholinergic interneurons arising from the LGE express the ISL1 LIM homeodomain transcription factor that is required for their survival and differentiation (Elshatory and Gan, 2008; Wang and Liu, 2001). Immunofluorescence analysis revealed the widespread presence of ISL1/2^+^ neurons surrounding neuroepithelial structures in mutant, but not in control, organoids (Figures 2Q and 2R). The MGE and CGE also give rise to GABAergic interneurons that migrate from their birthplace in the ventral telencephalon to the cortex. MGE-derived interneurons are characterized by NKX2.1 or somatostatin (SST) expression, while CGE interneurons are positive for COUP-TFII. Neurons expressing these markers were found in *INPP5E*^D477N/D477N^ organoids (Figures 2S–2X). Thus, mutant organoids lack dorsal telencephalic cell types but express a number of ventral telencephalic progenitor and neuron markers. This expression profile is consistent with the idea that the *INPP5E*^D477N/D477N^ mutation leads to a ventralization of cortical organoids.

### Sonic hedgehog signaling is up-regulated in *INPP5E*^D477N/D477N^ organoids

**Figure 3:**
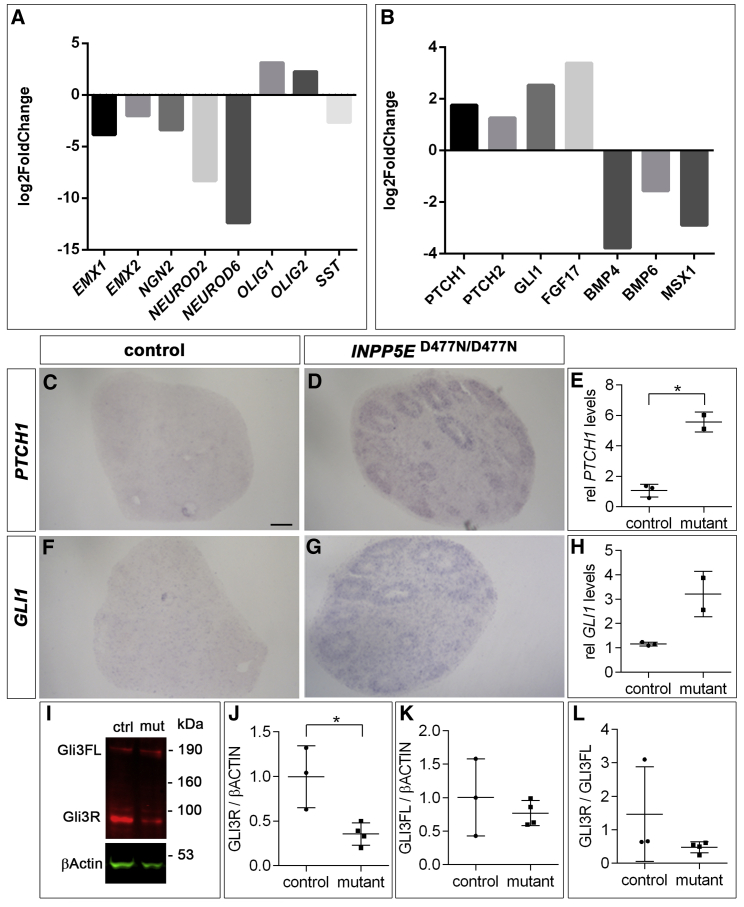
SHH signaling is up-regulated in *INPP5E*^D477N/D477N^ organoids (A and B) Examples of differentially expressed dorsal and ventral (A) and signaling factor genes (B) with indicated log2Fold changes in D24 organoids. (C, D, F, and G) *In situ* hybridization to detect *PTCH1* and *GLI1* expression in D39 control and *INPP5E* mutant organoids. (E and H) Representative example of qRT-PCR analyses showing *PTCH1* (E) and *GLI1* (H) mRNA expression relative to *ATP5* at D24. (I) GLI3 western blot on D24 organoid tissue revealed the GLI3 full-length (FL) and repressor (R) forms. (J–L) Quantification of GLI3 western blot. GLI3R levels are decreased in *INPP5E*^D477N/D477N^ organoids, while GLI3FL levels and the GLI3R/GLI3FL ratio (L) are not affected. All statistical data are presented as means ± SD; unpaired t tests with Welch’s correction (n = 3 for control and n = 2 for mutant; E and H); unpaired t tests (J and K); Mann-Whitney test (L) with n = 3 for control and n = 4 for mutant; ^∗^p < 0.05. Scale bar, 100 μm (C). See also Figures S4 and S5.

To identify the mechanisms behind this ventralization, we performed a bulk mRNA sequencing (mRNA-seq) experiment of D24 organoids. Analyzing control organoids confirmed the expression of markers specific for the telencephalon, but thalamus, midbrain, hindbrain, and spinal-cord markers were hardly expressed, if at all (Figure S4). A comparison between control and mutant organoids revealed no change in the transcription levels of the pan-telencephalic marker *FOXG1* and in *PAX6* expression but a down-regulation of the dorsal telencephalic progenitor markers *EMX1*, *EMX2*, and *NGN2* and of *NEUROD2* and *NEUROD6*, labeling newly born cortical projection neurons (Figure 3A). In contrast, the early ventral markers *OLIG1* and *OLIG2*, whose expression precedes that of *DLX2* and *ASCL1* (Hansen et al., 2013), were up-regulated. We also investigated the expression of signaling molecules and their downstream targets in the mRNA-seq dataset and noted an up-regulation of the SHH target genes *PTCH1* and *GLI1* and a down-regulation of *BMP4* and *BMP6* and their *MSX1* target gene (Figure 3B). Since SHH promotes ventral telencephalic development and represses dorsal cell fate acquisition in mice (Rallu et al., 2002b) and human organoids (Bagley et al., 2017; Birey et al., 2017), and since cilia are important regulators of SHH signaling, we hypothesized that the ventralized phenotype of *INPP5E*^D477N/D477N^ organoids originated from an up-regulation of SHH signaling. To test this hypothesis, we first examined the expression of the SHH target genes *PTCH1* and *GLI1* using *in situ* hybridization and found expression of both markers in the neuroepithelial rosettes of the mutant organoids, but not in control organoids (Figures 3C, 3D, 3F, and 3G). We confirmed this finding in quantitative RT-PCR experiments that showed a 5.3-fold and 2.8-fold increase in *PTCH1* and *GLI1* expression, respectively, although the increase in *GLI1* transcription was not statistically significant (Figures 3E and 3H). In addition to their role in activating the GLI transcription factors in the presence of SHH, cilia are critical for processing the GLI3 full-length protein (GLI3FL) to form the GLI3 repressor (GLI3R). Western blot analyses of organoid protein extracts revealed decreased GLI3R expression levels, while GLI3FL levels and the GLI3R/GLI3FL ratio were not affected (Figures 3I–3L and S5). Taken together, these data suggest an increase in SHH signaling coinciding with a reduced formation of GLI3R in *INPP5E*^D477N/D477N^ organoids.

### SHH signaling is necessary and sufficient to ventralize cortical organoids

**Figure 4:**
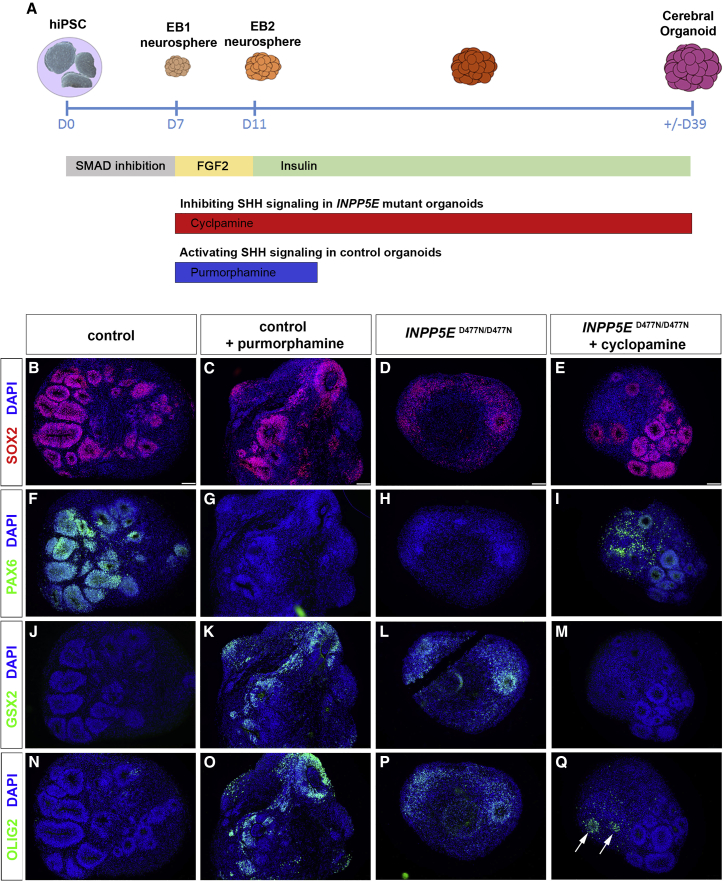
SHH signaling is necessary and sufficient to ventralize cortical organoids (A) Experimental protocol to either block or ectopically activate SHH signaling in cortical organoids. (B–Q) Organoids stained with the indicated antibodies after treatment with purmorphamine (C, G, K, and O) and cyclopamine (E, I, M, and Q). (B–E) SOX2 expression revealed neuroepithelia. (F–I) PAX6 expression is reduced in purmorphamine-treated control organoids but up-regulated after inhibiting SHH signaling in *INPP5E* mutant organoids. (J–Q) GSX2 and OLIG2 expression occurs in only a few cells in control organoids (J and N) but is up-regulated after activating SHH signaling (K and O) or in untreated mutant organoids (L and P). (M and Q) Cyclopamine treatment inhibits GSX2 and OLIG2 expression in mutant organoids except for a few cells (arrows in Q). Scale bars, 100 μm (B–E).

We next tested the role of the augmented SHH signaling in the induction of ventral marker gene expression in *INPP5E*^D477N/D477N^ mutant organoids and repeated the organoid experiment but added the SHH antagonist cyclopamine to the culture medium on day 7 for the remainder of the organoid culture to block the constitutive SHH signaling caused by the *INPP5E* mutation (Figure 4A). In a separate experiment, we also investigated whether activating SHH signaling was sufficient to induce a ventralization in cortical organoids derived from control iPSCs and treated control organoids after neural induction from day 7 for 1 week with the SHH agonist purmorphamine (Figure 4A) as described previously (James et al., 2021; Livesey et al., 2016).

Day 39 organoids from both experiments were harvested and stained for SOX2 to identify neuroepithelial structures (Figures 4B–4E) and for various dorsal and ventral telencephalic progenitor markers. These analyses first confirmed the expression of PAX6 and the absence of the ventral markers GSX2 and OLIG2 in control untreated organoids (Figures 4F, 4J, and 4N). In contrast, these ventral markers were up-regulated with a concomitant down-regulation of PAX6 in control organoids treated with purmorphamine (Figures 4G, 4K, and 4O), suggesting that activation of SHH signaling was sufficient to ventralize cortical organoids. As described above, *INPP5E*^D477N/D477N^ mutant organoids raised under control conditions also lacked PAX6 expression and showed an up-regulation of GSX2 and OLIG2 (Figures 4H, 4L, and 4P). Cyclopamine treatment, however, led to a mild up-regulation of neuroepithelial PAX6 (Figure 4I). Moreover, GSX2 and OLIG2 expression was largely absent, with a few neuroepithelial rosettes still expressing OLIG2 (Figures 4M and 4Q). Taken together, this staining pattern indicates that cyclopamine treatment confers a partial rescue of the ventralization caused by the *INPP5E* mutation.

### TULP3, GPR161, and IFT144 are enriched in the *INPP5E*^D477N/D477N^ mutant cilium

Next, we aimed to identify the molecular mechanisms that led to perturbed SHH signaling in the *INPP5E* mutant organoids. The INPP5E enzyme hydrolyzes the 5-phosphate from PI(4,5)P2 to produce PI(4)P. Consistent with INPP5E’s axonemal localization, PI(4)P levels were enhanced in the axoneme, while loss of *INPP5E* function led to PI(4,5)P_2_ accumulation in the axonemal membrane and at the ciliary tip and to altered hedgehog signaling (Chavez et al., 2015; Constable et al., 2020; Garcia-Gonzalo et al., 2015). Direct detection and distinction of phosphoinositides is technically difficult due to their fluidity and lipophilic characteristics. Therefore, we stained for the PI(4,5)P2 binding protein TULP3 as an indirect readout of PI(4,5)P2 distribution (Chavez et al., 2015; Constable et al., 2020; Garcia-Gonzalo et al., 2015) and studied the proportion of TULP3^+^ cilia and TULP3 expression levels in the axoneme by double immunofluorescence for TULP3 and the axonemal marker ARL13B. In control organoids, a small proportion of cilia were positive for TULP3, but TULP3 expression was detected in the majority of *INPP5E*^D477N/D477N^ mutant cilia (Figure S6). Moreover, the TULP3/ARL13B intensity ratios showed a significant increase in *INPP5E*^D477N/D477N^ organoids compared with controls (Figure S6).

As TULP3 recruits the SHH antagonist GPR161 and IFT-A components to the cilium (Chavez et al., 2015; Garcia-Gonzalo et al., 2015), we determined whether the increase in TULP3 in *INPP5E*^D477N/D477N^ organoids coincided with an enrichment of GPR161 and IFT144 in the cilium. Double immunofluorescence labeling for ARL13B and GPR161 or IFT144 revealed an increase in the proportion of GPR161^+^ and IFT144^+^ cilia and higher GPR161/ARL13B and IFT144/ARL13B intensity ratios in *INPP5E* mutant organoids (Figure S6). Taken together, these findings indicate an enrichment of TULP3, GPR161, and IFT144 in the *INPP5E*^D477N/D477N^ axoneme, consistent with a higher amount of PI(4,5)P2 in the axonemal membrane due to an impairment of INPP5E phosphatase activity.

### Increased ciliary expression of SMO and GLI2 in *INPP5E*^D477N/D477N^ cilia

**Figure 5:**
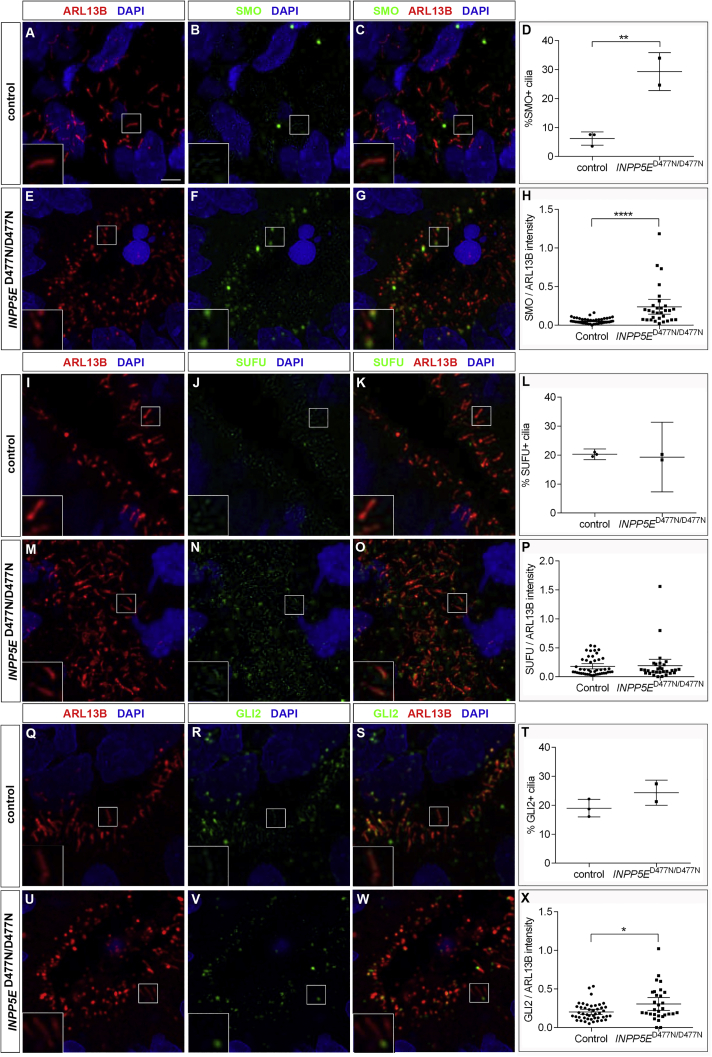
Ciliary expression of SHH signaling components Control and *INPP5E*^D477N/D477N^ organoids were immunostained with the indicated markers. (A–H) SMO was expressed in a higher proportion of cilia and at higher levels in *INPP5E*^D477N/D477N^ organoids. (I–P) There were no significant changes either in the proportion of positive cilia or in the expression levels for SUFU. (Q–X) GLI2 accumulated in mutant cilia. Statistical data are presented as means ± 95% confidence intervals (CIs); unpaired t tests (D, L, and T) and Mann-Whitney tests (H, P, and X); n = 3 (control) and n = 2 (mutant) lines for (D), (L), and (T); n = 45 (control) and n = 30 (mutant) cilia from three and two different lines, respectively (H, P, and X); ^∗^p < 0.05; ^∗∗^p < 0.01; ^∗∗∗∗^p < 0.0001. Scale bar, 2.5 μm. See also Figures S6 and S7.

To examine the effect of INPP5E on SHH signaling, we investigated the expression and localization of SHH signaling components. SMO, the main cellular transducer of hedgehog signals, accumulates in the cilium as a response to pathway activation and initiates the downstream signaling cascade (Corbit et al., 2005; Rohatgi et al., 2007). To compare SMO expression in the cilium, organoids were double stained for SMO and ARL13B. This revealed a significantly increased proportion of SMO^+^ cilia and SMO/ARL13B intensity ratios in *INPP5E*^D477N/D477N^ organoids (Figures 5A–5H), indicating enhanced SHH signaling.

We also studied ciliary expression of SUFU and of GLI2, the main repressor and transcriptional activator of SHH signaling, respectively. Activation of hedgehog signaling leads to both proteins entering the cilium and accumulating at the ciliary tip (Corbit et al., 2005; Haycraft et al., 2005; Wen et al., 2010). Given these well-characterized changes in the distribution of SUFU and GLI2 proteins, we determined their localization in control and *INPP5E*^D477N/D477N^ organoids. We did not find significant changes in the proportion of SUFU^+^ cilia or in SUFU expression levels but an increase in GLI2 ciliary expression (Figures 5I–5X). Collectively, these data indicate an enrichment of SMO and GLI2 in the cilium of *INPP5E* mutant RGCs but no change for SUFU.

### Transition zone and protein transport in cilia

To identify the molecular basis for the enrichment of both positive (SMO) and negative (GPR161) regulators of SHH signaling in the mutant ciliary axoneme, we became interested in the transition zone (TZ) that controls entry and exit of proteins in and out of the cilium, respectively (Garcia-Gonzalo et al., 2011; Reiter et al., 2012). Interestingly, *Inpp5e* is required for the molecular organization and maturation of the TZ in mice and flies (Dyson et al., 2017; Gupta et al., 2018). To address the possibility that *INPP5E* might also be necessary for the integrity of the transition zone in human cortical organoids, we performed immunofluorescence stainings for RPGRIP1L, TCTN1, and TMEM67. RPGRIP1L coordinates TZ assembly (Wiegering et al., 2018), while TCTN1 and TMEM67 encode components of the MKS complex (Garcia-Gonzalo et al., 2011). Our immunolabeling revealed all three proteins at the TZ of control aRGC cilia with low expression levels in the ciliary axoneme. The localization of these proteins at the TZ was not affected, but axoneme expression of RPGRIP1L and TCTN1 was slightly reduced but increased for TMEM67 (Figure S7). These findings suggest subtle changes at the mutant TZ.

**Figure 6:**
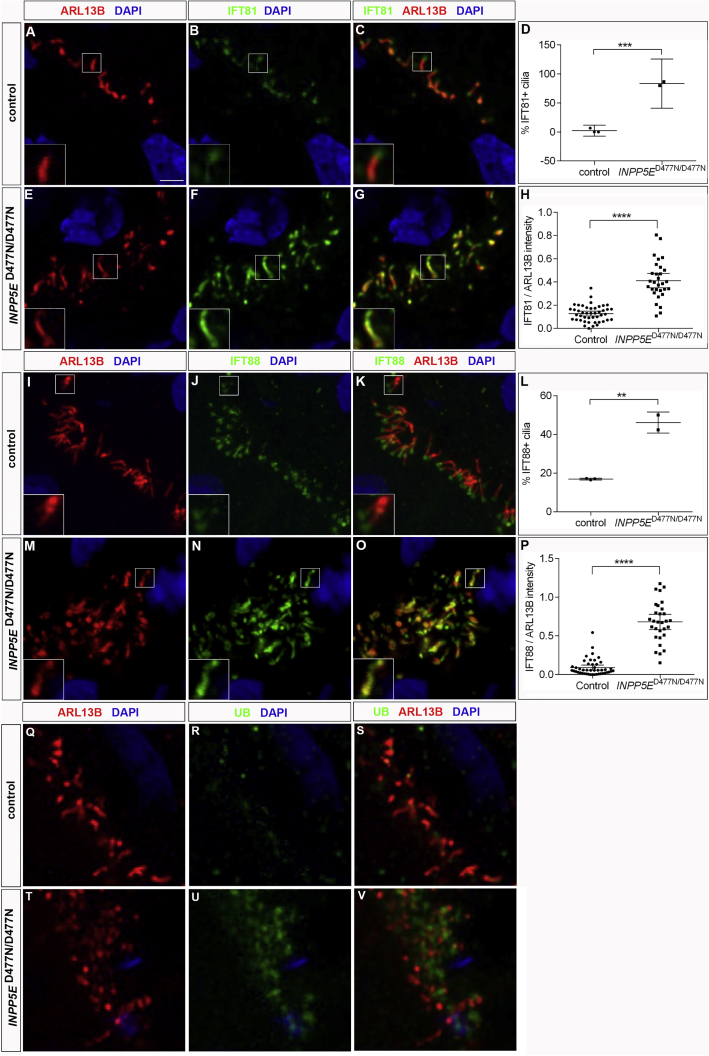
IFT-B proteins in *INPP5E*^D477N/D477N^ organoids Control and *INPP5E*^D477N/D477N^ organoids were immunostained with the indicated markers. (A–P) IFT81 and IFT88 were expressed in a higher proportion of ciliary axonemes and at increased levels. (Q–V) UB expression increased at the base, but not in the axoneme, of *INPP5E*^D477N/D477N^ cilia. Statistical data are presented as means ± 95% CIs; unpaired t tests (D and L); unpaired t test with Welch’s correction (H) and Mann-Whitney tests (P); n = 3 (control) and n = 2 (mutant) lines for (D), (L), and (T); n = 45 (control) and n = 30 (mutant) cilia from three and two different lines, respectively (H, P, and X); ^∗∗^p < 0.01; ^∗∗∗^p < 0.001; ^∗∗∗∗^p < 0.0001. Scale bar, 2.5 μm. See also Figures S6 and S7.

To further explore the mechanisms underlying SMO accumulation, we investigated the expression and ciliary localization of IFT proteins, as defective IFT can result in increased SMO localization in cilia (Wang et al., 2021). As the IFT-A component IFT-144 accumulated in mutant cilia (Figure S6), we analyzed whether the localization of IFT-B proteins is also affected. In control cilia, IFT81 and IFT88 were found at high levels at the ciliary base with lower expression levels in the axoneme and at the ciliary tip. In mutant cilia, however, we detected high levels of IFT81 or IFT88 co-expression with ARL13B in the axoneme in an increased proportion of cilia (Figures 6A–6P). In addition to intact intraflagellar transport, ubiquitination of activated G protein coupled receptors (GPCRs) is required for their BBSome-mediated exit from the cilium (Desai et al., 2020; Shinde et al., 2020). Investigating ubiquitin (UB) distribution revealed an aggregation of UB at the mutant ciliary base, but not in the axoneme (Figures 6Q–6V). Taken together, these findings suggest that the transport of activated SMO out of the cilium might be affected in mutant progenitor cells.

## Discussion

Understanding the emerging roles of primary cilia in the pathogenesis of neurodevelopmental disorders requires a better knowledge of their roles in physiological human brain development. Here, we investigated the consequences of inactivating the ciliary *INPP5E* gene on forebrain development in a human cortical organoid model and showed that it is essential for D/V patterning. In the absence of functional *INPP5E*, organoids formed ventral telencephalic progenitors and neurons. This phenotype was caused by an up-regulation of SHH signaling that is necessary and sufficient to induce this ventralization. The *INPP5E* mutation also led to an accumulation of SMO, GLI2, GPR161, and several IFT proteins in the cilium and of ubiquitinated proteins at the ciliary base. Taken together, these findings implicate *INPP5E* as a negative regulator of SHH signaling in human cortical organoids. They also emphasize the importance of cilia for early human corticogenesis and offer insights into disease mechanism underlying neurodevelopmental disorders.

### Cilia and dorsal and ventral patterning of the human telencephalon

While we are gaining an increasing understanding of mammalian nervous system development from the study of model organisms, this knowledge might not be directly applicable to the developing human brain, given its dramatically increased size and its species-specific cellular features (Florio and Huttner, 2014; Hodge et al., 2019). Human organoids represent an excellent experimental system to bridge this knowledge gap (Arlotta and Pasca, 2019). Here, we applied cortical organoids to investigate potential roles of primary cilia in an early step in telencephalic development and its subdivision in specific dorsal and ventral territories from which the cortex and basal ganglia develop, respectively. These organoids form neuroepithelial structures with primary cilia projecting from the apical surface of progenitor cells into the ventricular lumen, reflecting the arrangement in the forebrain of a PCW 8 human embryo. Moreover, inactivating the phosphatase activity of *INPP5E*, which is critical for ciliary biology and cilia-mediated signaling, resulted in a striking formation of ventral telencephalic progenitors and neurons due to a prolonged activation of SHH signaling. This ventralization is more severe and robust than the mild defects at the pallial/subpallial boundary and the transient spinal cord patterning defect in *Inpp5e* mutant mice (Constable et al., 2020; Hasenpusch-Theil et al., 2020). The greater severity could be due to an absence of dorsalizing BMP signals (Furuta et al., 1997). Alternatively, human and mouse forebrain may follow different paths to establish distinct dorsal and ventral telencephalic domains. It has been suggested that, during D/V patterning of human forebrain, neuroectoderm first acquires forebrain dorsal fate by default, while the acquisition of ventral telencephalic fates requires the repression of dorsal transcription factors through SHH signaling (Chi et al., 2017; Zhang et al., 2010). In line with this model, neural organoids exhibit dorsal forebrain identity in the absence of any exogenous signals. This idea is also supported by our observation that, unlike other dorsal telencephalic markers, *PAX6* expression was still unaltered in D24 *INPP5E* mutant organoids, while it was lost later at D39. Moreover, our findings and that of others that the activation of SHH signaling either genetically or pharmacologically (Bagley et al., 2017; Birey et al., 2017) induces ventral cell fates in organoids provide further evidence for this hypothesis. In contrast, in the mouse mutual repression between (1) Wnt/Bmp/Shh signaling and (2) the Pax6/Nkx2.1 transcription factors underlies the specification of dorsal and ventral cell fates (Gunhaga et al., 2003; Sussel et al., 1999). Regardless of this difference, the ventralization phenotype in *INPP5E* organoids places primary cilia through their control of SHH signaling at the center of this patterning process. This finding also has implications for our understanding of neurodevelopmental disorders. It is unknown whether D/V patterning of the telencephalon is affected in JS; these patients often show cortical malformations, including polymicrogyria (Valente et al., 2014), that could be caused by overactive SHH signaling in outer RGCs (Matsumoto et al., 2020; Wang et al., 2016). Moreover, *INPP5E* JS mutations are hypomorphic point mutations resulting in reduced phosphatase activity (Bielas et al., 2009; Jacoby et al., 2009). Hence, our organoids using the *INPP5E*^D447N/D477N^ mutation can serve as a paradigm to investigate the effects of *INPP5E* JS patient-specific mutations on neural development. Finally, a widely regarded hypothesis proposes that an imbalance between excitation and inhibition (E/I imbalance) underlies phenotypical manifestation of many neurodevelopmental disorders (Bourgeron, 2009; Kepecs and Fishell, 2014; Marin, 2012). As a consequence of altered SHH signaling, mutations in ciliary genes could alter the size of the dorsal and ventral progenitor domains and thereby the relative proportions of glutamatergic projection neurons and GABAergic inhibitory neurons produced in these different territories. This scenario provides a different disease mechanism to previously described roles of primary cilia in controlling the tangential migration of murine cortical interneurons (Baudoin et al., 2012; Higginbotham et al., 2012), their morphology, and synaptic connectivity (Guo et al., 2017). Taken together, these studies illustrate several potential ways how defects in primary cilia could affect the E/I balance crucial for proper cortical functioning.

### Mechanism of up-regulated SHH signaling in *INPP5E*^D477N/D477N^ organoids

Besides providing insights into a fundamental process in human forebrain development, our study also sheds light on the molecular mechanisms by which *INPP5E* controls SHH signaling. Previous analyses established tissue-specific roles for *INPP5E* as a positive or negative regulator of SHH signaling (Chavez et al., 2015; Constable et al., 2020; Garcia-Gonzalo et al., 2011). In murine adult neural stem cells and IMCD3 cells, *Inpp5e* enables Shh signaling by limiting the ciliary levels of the Hh inhibitors Tulp3 and Gpr161 (Chavez et al., 2015; Garcia-Gonzalo et al., 2011). TULP3 and GPR161 levels were also increased after *Inpp5e* inactivation in the developing ventral spinal cord (Constable et al., 2020) and in our organoids. Interestingly, in both systems, SHH signaling was augmented despite the increase of the TULP3 and GPR161 repressors. The output of SHH signaling is ultimately determined by the relative levels of GLI activator and repressors. Accordingly, Constable et al. (2020) proposed a decrease in GliR production and a concomitant lower GliR/GliA ratio, but their western blot data did not support this hypothesis, probably due to the use of whole-embryo extracts rather than tissue-specific samples (Constable et al., 2020). The human organoid samples, however, consisted largely of cortical tissue, and our analysis revealed decreased GLI3R levels, whereas GLI3FL levels and the GLI3R/GLI3FL ratio were not significantly altered. Concomitantly, we also noted an accumulation of GLI2 in mutant cilia. These findings suggest that cilia-mediated control of GLI3R formation and GLI2 activation are critical for dorsal telencephalic development in human organoids and are also consistent with the activation and transformation model of D/V patterning the human telencephalon (Chi et al., 2017).

**Figure 7:**
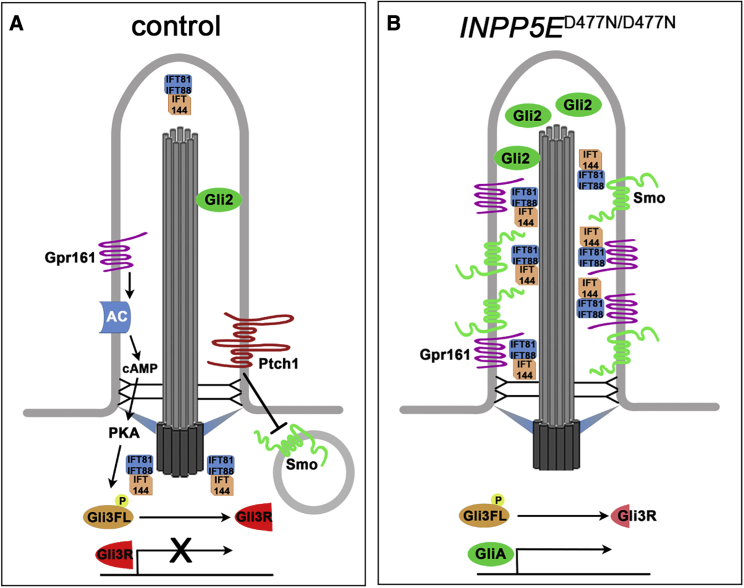
Schematic summary of the changes in the activity and localization of SHH signaling components identified in *INPP5E* mutant cortical organoids (A) In control organoids, SMO ciliary levels are low and the GLI3 repressor form (GLI3R) suppressing SHH signaling predominates. (B) In *INPP5E* mutant organoids, the ciliary localization of several negative and positive regulators of SHH signaling is disturbed. SMO, GLI2, GPR161, and several IFT proteins accumulate in the cilium. GLI3R levels are reduced, and SHH target genes are activated.

This leaves the question how loss of *INPP5E* phosphatase activity leads to defective GLI3 processing. While the accumulation of SMO and GLI2, the main activators of the pathway, is consistent with increased SHH signaling, cilia also contained augmented levels of the SHH repressor GPR161 (Figure 7). Interestingly, GPR161 forms a module with a regulatory subunit of PKA that, by amplifying cyclic AMP (cAMP) signals, modulates PKA activity but exits the cilium in the presence of SHH (May et al., 2021). Hence, GPR161’s accumulation in the *INPP5E*^D477N/D477N^ cilium could lead to prolonged PKA activation, GLI3 hyperphosphorylation, and increased proteolytic degradation. This idea is supported by our observation of polyubiquitinated protein aggregates at the ciliary base. Alternatively, the simultaneous presence of both activators and repressors without SHH pathway stimulation was previously noted (Shinde et al., 2020) and may indicate that the mechanisms that control transport and exit of these molecules out of the cilium were not operating properly. Unlike previous observations (Dyson et al., 2017), *INPP5E* mutant cilia showed normal expression of RPGRIP1L, TCTN1, and TMEM67 at the TZ. Alternatively, ubiquitination is required to remove SMO from the cilium in an IFT27- and BBSome-dependent manner (Desai et al., 2020; Shinde et al., 2020), but despite the SMO accumulation, we did not observe an increase in ciliary protein ubiquitination within the cilium. These observations suggest that defects in the TZ or in protein ubiquitination are unlikely to underlie the augmented SHH signaling. In contrast, components of the IFT-A and B machinery accumulated in mutant cilia. Defects in IFT are known to cause abnormal SMO localization (Eguether et al., 2014; Keady et al., 2012; Liew et al., 2014; Yang et al., 2015). Abnormal transport could also be caused by a defect in the BBSome (Hey et al., 2021). Overall, these findings indicate that reduced GLI3R levels in combination with defects in protein transport are likely mechanisms leading to increased SHH signaling and to the subsequent ventralization of *INPP5E*^D477N/D477N^ organoids.

### Limitations of the study

Using double SMAD inhibition guides organoid development toward a cortical fate with high consistency, but to a certain degree, neural progenitor and neurons typical of other brain regions are present. The extent of this heterogeneity and how it effects organoid differentiation is difficult to estimate from immunostainings and bulk mRNA sequencing. Single-cell RNA-seq experiments will shed light on this issue and enable cell-type-specific comparisons between control and mutant organoids.

While we show that ectopic SHH signaling is sufficient and necessary for ventralizing control and *INPP5E* mutant cortical organoids, respectively, additional ciliary-mediated signaling pathways are likely to be affected. For example, our RNA-seq analysis identified a down-regulation of *BMP4/6* expression that might act in combination with activated SHH signaling. Future pharmacological approaches to inhibit and activate BMP signaling in control and *INPP5E* mutant organoids, respectively, are required to further unravel the mechanisms that underlie D/V patterning of the human forebrain.

## STAR★Methods

### Key resources table

**Table:** 

REAGENT or RESOURCE	SOURCE	IDENTIFIER
**Antibodies**		

Mouse anti-Arl13b (clone N295B/66) (monoclonal)	UC Davis/NIH NeuroMab Facility	Cat# 75-287; RRID: AB_2341543
Rabbit anti-ARL13B (polyclonal)	Proteintech	Cat# 17711-1-AP; RRID: AB_2060867
Rabbit anti-COUP-TFII (polyclonal)	Provided by M Studer	
Rat anti-Ctip2 (monoclonal)	Abcam	Cat# ab18465; RRID: AB_2064130
Guinea pig anti-DLX2 (polyclonal)	Bioacademica	Cat# 74-116
Rabbit anti-EMX1 (polyclonal)	Edoardo Boncinelli	Briata et al. (1996)
Rabbit anti-FOXG1 (polyclonal)	Abcam	Cat# ab18259; RRID: AB_732415
Guinea pig anti-GLI2 (polyclonal)	Jonathan Eggenschwilter	Cho et al. (2008)
Mouse anti-Polyglutamylation Modification (clone GT335)	AdipoGen	Cat# AG-20B-0020; RRID: AB_2490210
Rabbit anti-GPR161 (polyclonal)	Proteintech	Cat# 13398-1-AP; RRID: AB_2113965
Rabbit anti-GSX2 (polyclonal)	Millipore	Cat# ABN162; RRID: AB_11214376
Rabbit anti-IFT81 (polyclonal)	Proteintech	Cat# 11744-1-AP; RRID:AB_2121966
Rabbit anti-IFT88 (polyclonal)	Proteintech	Cat# 13967-1-AP; RRID:AB_2121979
Rabbit anti-IFT144/WDR19 (polyclonal)	Proteintech	Cat#13647-1-AP; RRID: AB_10598484
Rabbit anti-INPP5E (polyclonal)	Proteintech	Cat# 17797-1-AP; RRID: AB_2167120
Mouse anti-ISL1/2 (monoclonal)	DSHB	Cat# 39.4D5; RRID: AB_2314683
Mouse anti-NKX2.1/TTF1 (monoclonal) (clone 8G7G3/1)	Abcam	Cat# ab3186
Rabbit anti-OLIG2 (polyclonal)	Millipore	Cat# AB9610; RRID: AB_570666
Rabbit anti-PAX6 (polyclonal)	Biolegend	Cat# 901301; RRID: AB_2565003
Rabbit anti-RPGRIP1L (polyclonal)	Proteintech	Cat#55160-1-AP; RRID: AB_10860269
Rabbit anti-SMO (polyclonal)	Proteintech	Cat# 20787-1-AP; RRID: AB_2878740
Rabbit anti-SOX2 (monoclonal)	Abcam	Cat# ab92494; RRID: AB_10585428
Rabbit anti-SST (Somatostatin-14)	Peninsula Laboratories	Cat# T-4102.0400; RRID: AB_518613
Rabbit anti-SUFU (polyclonal)	Proteintech	Cat# 26759-1-AP; RRID: AB_2880625
Rabbit anti-TBR1 (polyclonal)	Abcam	Cat# ab31940; RRID: AB_2200219
Rabbit anti-TBR2 (polyclonal)	Abcam	Cat# ab23345; RRID:AB_778267
Rabbit anti-TCTN1 (polyclonal)	Proteintech	Cat#15004-1-AP; RRID: AB_10644442
Rabbit anti-MSK3/TMEM67 (polyclonal)	Proteintech	Cat# 13975-1-AP; RRID: AB_10638441
Mouse anti-gammaTUB (monoclonal) (clone GTU-88)	Sigma-Aldrich	Cat# T6557; RRID: AB_477584
Rabbit anti-TULP3 (polyclonal)	Proteintech	Cat# 13637-1-AP; RRID: AB_2211547
Mouse anti-multi Ubiquitin IgG1 clone FK2	MBL International	Cat# D058-3; RRID: AB_592937
Donkey anti-mouse IgG Cy2 (polyclonal)	Jackson ImmunoResearch Labs	Cat# 715-225-151; RRID: AB_2340827
Donkey anti-rabbit IgG Cy3 (polyclonal)	Jackson ImmunoResearch Labs	Cat# 711-165-152; RRID: AB_2307443
Goat anti-rat IgG Cy3 (polyclonal)	Jackson ImmunoResearch Labs	Cat# 112-165-003; RRID: AB_2338240
Goat anti-mouse IgG2b, Alexa Fluor 647 conjugated (polyclonal)	Innovative Research	Cat# A21242; RRID: AB_1500900
Goat anti-rat IgG Alexa Fluor 647 conjugated (polyclonal)	Molecular Probes	Cat# A-21247; RRID: AB_141778
Pig anti-rabbit IgG, biotinylated (polyclonal)	Dako	Cat# E0431
Streptavidin, Alexa Fluor 488 conjugate antibody	Molecular Probes	Cat# S32354; RRID: AB_2315383
Streptavidin, Alexa Fluor® 568 conjugate antibody	Thermo Fisher Scientific	Cat# S-11226; RRID: AB_2315774
DAPI (4′,6-Diamidino-2-Phenylindole, Dihydrochloride)	Thermo Fisher Scientific	Cat# D1306; RRID: AB_2629482
Mouse anti-TRA-1-60 (monoclonal)	Santa Cruz Biotechnology	Cat# sc-21705; RRID: AB_628385
Rabbit anti-NANOG (polyclonal)	Cell Signaling Technology	Cat# 3580; RRID: AB_2150399
Mouse anti-OCT3/4 (monoclonal)	Santa Cruz Biotechnology	Cat# sc-5279; RRID: AB_628051
Goat anti-rabbit IgG Alexa Fluor 488 conjugated (polyclonal)	Molecular Probes	Cat# A-11008; RRID: AB_143165
Goat anti-mouse IgM, Alexa Fluor 555 conjugated (polyclonal)l	Innovative Research	Cat# A21426; RRID: AB_1500929
Goat anti-rabbit IgG, biotinylated (polyclonal)	Agilent	Cat# E0432; RRID: AB_2313609
Mouse anti-β-Actin (clone AC-15) (monoclonal)	Abcam	Cat# ab6276; RRID:AB_2223210
Goat anti-h/m GLI3 (polyclonal)	R&D Systems	Cat# AF3690; RRID: AB_2232499
IRDye 680RD Donkey anti-Goat IgG	LI-COR Biosciences	Cat# 926-68074; RRID: AB_10956736
IRDye 800CW Donkey anti-Mouse IgG	LI-COR Biosciences	Cat# 925-32212; RRID: AB_2716622

**Biological samples**		

Human embryonic and fetal brain tissue	Human Developmental Biology Resource	www.hdbr.org

**Chemicals, peptides, and recombinant proteins**		

Essential 8™ Medium	Thermo Fisher Scientific	Cat#A1517001
Matrigel Basement Membrane Matrix High Concentration (HC)	Scientific Laboratory Supplies	Cat#354230
Matrigel	Corning	Cat#354248
DMEM/F12	Thermo Fisher Scientific	Cat#11330032
Fetal Calf Serum (FCS)	Thermo Fisher Scientific	Cat#12103C
L-glutamine	Thermo Fisher Scientific	Cat#25030024
Lipofectamine 2000	Thermo Fisher Scientific	Cat#STEM00001
T7 endonuclease I	New England Biolabs	Cat#M0302
Accutase	Stem Cell Technologies	Cat#0792207920
Rock Inhibitor (Y-27632)	Stem Cell Technologies	Cat#72302
Puromycin	Thermo Fisher Scientific	Cat#J67236.8EQ
GoTaq G2 DNA polymerase	Promega	Cat#M7845
ApoI-HF	New England Biolabs	Cat#R3566L
Antibiotic-Antimycotic	Thermo Fisher Scientific	Cat#15240062
Dispase	Thermo Fisher Scientific	Cat#17105041
Collagenase	Thermo Fisher Scientific	Cat#17104019
Iscove’s Modified Dulbecco’s Medium (IMDM)	Thermo Fisher Scientific	Cat#21980032
Ham’s F-12 Nutrient Mix	Thermo Fisher Scientific	Cat#21765029
BSA	Europa Bioproducts	Cat#EQBAC62
Chemically Defined Lipid Concentrate	Thermo Fisher Scientific	Cat#11905031
Monothioglycerol	Sigma	Cat#M6145
Human Insulin	Sigma	Cat#11376497001
Transferrin	Sigma	Cat#10652202001
N-acetyl cysteine	Sigma	Cat#A8199
Activin Inhibitor (SB431542)	R & D Systems	Cat#1614
LDN-193189	StraTech	Cat#S2618-SEL
Advanced DMEM/F12	Thermo Fisher Scientific	Cat#12634028
GlutaMAX™ Supplement	Thermo Fisher Scientific	Cat#35050038
N2 Supplement	Thermo Fisher Scientific	Cat#17502001
B27 Supplement	Thermo Fisher Scientific	Cat#17504001
Murine FGF-basic	PeproTech	Cat#450-33
Neurobasal™ Medium	Thermo Fisher Scientific	Cat#21103049
B27 Supplement Minus Vitamin A	Thermo Fisher Scientific	Cat#12587010
MEM Non-Essential Amino Acids Solution	Thermo Fisher Scientific	Cat#11140050
Purmorphamine CAS 483367-10-8-Calbiochem	Merk	Cat#540220
Cyclopamine, V.californicum	Millipore	Cat#239803
Superscript™ IV VILO™ Master Mix with ezDNase	Thermo Fisher Scientific	Cat#11766050
NuPAGE Tris-Acetate Mini gel (3-8%)	Life Technologies	Cat#EA0375

**Critical commercial assays**		

Amaxa P3 Primary Cell 4D-Nucleofector™ X Kit	Lonza	Cat#V4XP-3012
RNeasy Plus Micro Kit	Qiagen	Cat#74034
QuantiFast SYBR Green PCR Kit	Qiagen	Cat#204054

**Deposited data**		

RNAseq *INPP5e* organoids	This paper	EBI: E-MTAB-11437

**Experimental models: Cell lines**		

iPSC control line hPSC1	Mandy Johnstone	(Johnstone et al., 2019; Selvaraj et al., 2018; Vasistha et al., 2019)
iPSC control lines hPSC2 (male) (CS02iCTR-n1)	Cedars-Sinai	N/A
iPSC control lines hPSC3 (male) (CS25iCTR-18n2)	Cedars-Sinai	N/A
iPSC INPP5E^D477ND477N^ hPSM1 clone (1C2)	This paper	N/A
iPSC INPP5E^D477ND477N^ hPSM2 clone (2A6)	This paper	N/A
HEK 293	ATCC	https://www.atcc.org/products/crl-1573

**Oligonucleotides**		

gRNA 5′-CTGTGCGCCCGCCACTCAGG-3′	This paper	N/A
ssODN D447N for gene editing 5′GCCGCAGCGGACGTCACCACCCGCTTCGATGAGGTGTTC TGGTTTGGAAATTTCAACTTCAGGCTGAGTGGCGGGCGCAC AGTCGTGGACGCCCTCCTGTGCCAGGGCCTGGTGGTGGAC GTGCCGGCGCTGCTGCAGCACGACCAGCTCATCCGGGAGA TGCGGAAAGGTG3′	This paper	N/A
Inp D477N Fw 5′-GCGGTTCTTTAGCACGGTTA-3′	This paper	N/A
Inp D477N Rev 5′-CTCCTCATCTCCCTCCATG-3′	This paper	N/A
Oligonucleotides for CrisprCas9 off targets, see Table S1	This paper	N/A
Primers for ISH and qPCR, see Table S1	This paper	N/A

**Recombinant DNA**		

pSpCas9(BB)-2A-Puro (PX459)	(Ran et al., 2013)	RRID:Addgene_48139
pEGFP-Puro	(Abbate et al., 2001)	RRID:Addgene_45561
pBS hSHH (CT#401)	Cliff Tabin	RRID:Addgene_13996
pSpCas9(BB)-gRNA(INPP5E-D477N)-2A-Puro (PX459)	This paper	N/A

**Software and algorithms**		

Fiji (ImageJ)	https://imagej.net/Fiji	N/A
Image Studio Lite	http://www.licor.com/bio/products/software/impage_studio_lite	RRID:SCR_013715
GraphPad Prism 9	http://graphpad.com	RRID:SCR_002798
Adobe Photoshop (12.1)	https://www.adobe.com/products/photoshop.html	RRID:SCR_014199
CRISPR design tool	http://crispr.mit.edu	Broad Institute
Opticon Monitor software v1	Bio-Rad Laboratories, Inc.	N/A
Huygens Essential Software	https://svi.nl/HuygensSoftware	RRID:SCR_014237
STAR alignment	(Dobin et al., 2013)	https://www.ncbi.nlm.nih.gov/pubmed/23104886
Samtools	(Li et al., 2009)	http://samtools.sourceforge.net/
FeatureCounts	(Liao et al., 2014)	https://www.ncbi.nlm.nih.gov/pubmed/24227677
R Studio version 1.2.5033	Tim Lebedkov	https://www.npackd.org/p/rstudio/1.2.5033
Deseq2 version 1.30.1	(Love et al., 2014)	https://www.ncbi.nlm.nih.gov/pubmed/25516281

### Resource availability

#### Lead contact

Further information and requests for resources and reagents should be directed to and will be fulfilled by the Lead Contact, Thomas Theil (thomas.theil@ed.ac.uk).

#### Materials availability

Unique material generated in this study is available from the Lead contact with a completed Materials Transfer Agreement.

### Experimental model and subject details

The human embryonic material was provided by the Joint MRC/Wellcome Trust (grant# MR/R006237/1) Human Developmental Biology Resource (www.hdbr.org). The HDBR has ethical approval from the NHS Health Research Authority.

The human pluripotent stem cell-lines used in this study were obtained with full Ethical/Institutional Review Board approval by the University of Edinburgh and validated using standard methods including chromosomal analysis, pluripotency and absence of plasmid integration. The hPSC1 line was described previously (Johnstone et al., 2019; Selvaraj et al., 2018; Vasistha et al., 2019), the additional iPSC control lines hPSC2 (CS02; male) and hPSC3 (CS25; male) were obtained from Cedars-Sinai. Two *INPP5E* mutant lines, named hPSM1 (1C2) and hPSM2 (2A6) were newly established from the hPSC1 line (see below).

### Method details

#### Cell culture

iPSCs were continuously maintained in Essential 8™ medium (Gibco, ThermoFisher) on Matrigel® (Corning) coated 6-well plates. HEK 293 cells were cultured in DMEM/F-12 (Gibco, ThermoFisher) supplemented with 10% Foetal Calf Serum (FCS) (Gibco, ThermoFisher) and 2 mM L-glutamine (Gibco, ThermoFisher). All cell types were maintained at 37°C in a 5% CO_2_ atmosphere.

#### Gene editing by CRISPR/Cas9 Homology-Directed Repair

Generating the *INPP5E* D447N mutation was performed in the iPSC1 line. To ensure that this line did not contain unknown *INPP5E* mutations, the targeted exon and flanking sequences were sequenced prior to gene editing. gRNAs were designed using an online CRISPR design tool (http://crispr.mit.edu) and were cloned into the pSpCas9(BB)-2A-Puro (PX459) plasmid (Addgene: #48139). To test gRNA efficiency a T7 endonuclease assay was performed. gRNA constructs were transfected with Lipofectamine 2000 (Invitrogen, ThermoFisher) into HEK 293 cells, which had been seeded 16-24 hours prior to transfections. Cells were harvested 48 hours post-transfection and genomic DNA was extracted using the Wizard® SV Kit (Promega). Genomic targeting efficiency for each gRNA was determined through annealing and digestion with T7 Endonuclease I (NEB: #M0302) of PCR products flanking the INPP5E D447N target site. gRNA 5′-CTGTGCGCCCGCCACTCAGG-3′ was determined as optimal for use in gene-editing. iPSCs at 70-80% confluence were dissociated into single cells with Accutase (Stemcell Technologies) and 8x10^5^ cells were electroporated with 2 μg Cas9-sgRNA plasmid (Addgene: #48139), 1 μg pEGFP-Puro and 200μM (6.6 μg) 180nt single-stranded DNA oligonucleotide donor template (ssODN) (PAGE-purified; Integrated DNA Technologies), using the P3 Primary Cell 4D-Nucleofector™ X Kit (Lonza), (program CA-137) on aLonza 4D-Nucleofector™ X Unit (Lonza) according to manufacturer’s guidelines. Transfected cells were resuspended in pre-warmed Essential 8™ medium supplemented with 10 μM ROCK-inhibitor (Y-27632, Stemcell Technologies) and seeded into two wells of a Matrigel® coated 6-well plate. Selection with 1 μg/ml puromycin (ThermoFisher) was commenced 24 hours post-nucleofection and continued for 24 hours. Cells were grown to confluence and passaged at low density (5x10^3^ and 1x10^4^), as single cells onto Matrigel® coated 10 cm dishes in Essential 8™ medium with 10 μM Y27632. After 8-10 days single-cell derived colonies were isolated and transferred to a Matrigel® coated 96-well plate. Duplicate plates were made for maintenance and restrictionfragment-length polymorphism (RFLP) screening. When cells for genotyping reached confluence, crude genomic DNA lysates were prepared by adding 50μl Cell Lysis Buffer (0.45% NP-40 substitute, 0.45% Tween-20, 0.2 mg/ml Proteinase K, 0.05x PCR Buffer in dH_2_O) and incubated at 55°C for 2 hours, followed by 10 minutes at 95°C. Amplicons flanking the targeting site were amplified with the following primers: Inp D447N Fw GCGGTTCTTTAGCACGGTTA and Inp D447N Rev: CTCCTCATCTCCCTCCATG using GoTaq G2 polymerase (Promega). PCR protocol: 95°C for 2 minutes; 35 cycles of 95°C for 15 seconds, 60°C for 30 seconds, 72°C for 30 seconds; and a final extension at 72°C for 5 minutes. PCR products were digested with ApoI-HF (New England Biolabs) and run on a 2% TAE agarose gel. Clones identified as carrying ApoI restriction site were evaluated for introduction of D447N mutation through Sanger sequencing (Source Bioscience). The top 5 candidates for off-target effects identified with an online tool (http://crispr.mit.edu) were sequenced using oligonucleotides as summarized in Table S1. Successfully edited clones were expanded and re-sequenced and assessed for chromosomal abnormalities G-banding karyotype analysis. Quality control tests were performed after clonal passage 10 and included immunocytochemistry with a panel of antibodies to pluripotency markers (TRA-160, OCT3/4, NANOG) (Figure S1). Aneuploidy BOBS assay (karyotyping) was done by TDL Genetics, London (Data S1).

#### Generation of cerebral organoids

Cerebral organoids were generated and maintained according to a modified Lancaster protocol (Lancaster et al., 2013) as described recently (Johnstone et al., 2019). This protocol evades the generation of embryoid bodies and goes straight to making neurospheres by dual-SMAD inhibition (Chambers et al., 2009). hiPSCs were cultured in Matrigel® Matrix coated 6-well plates in Essential 8™ Basal Medium supplemented with Antibiotic-Antimycotic (Invitrogen). The cells were grown for an average of seven days and lifted when cultures reached around 80% confluency with distinct, well defined hiPSC colonies. The colonies were lifted with a 1:1 Dispase/Collagenase enzyme mix (1mg/ml; Gibco and 2 mg/ml; Gibco) and resuspended in 10 ml of Phase 1 medium (1:1 IMDM (Invitrogen): Ham’s F-12 Nutrient Mix (Invitrogen); 5g/l BSA Cohn fraction V (Europa-bioproducts), 1/100 Chemically Defined Lipid Concentrate (Invitrogen); 1/25,000 Monothioglycerol (Sigma); 7μg/ml human Insulin (Sigma), 1/2000 Transferrin (Sigma), 1/100 Antibiotic-Antimycotic; 1mM N-acetyl cysteine (Sigma); 10μM Activin Inhibitor (SB 431542; R & D systems) and 0.1μM LDN (Stratech). From this point onwards, cells were cultured in suspension on an orbital shaker at 45 rpm in a cell culture CO_2_ incubator at 37⁰C and 5% CO_2_. After seven days the colonies were transferred to EB1 medium containing Advanced DMEM/F12 (Invitrogen) supplemented with 1/100 Antibiotic-Antimycotic, 1/100 GlutaMAX™-I Supplement (Invitrogen), 1/100 N2 Supplement (Invitrogen), 1/200 B27 Supplement (100 M; Invitrogen) and 2.5 ng/ml Murine FGF-basic (Peprotech). After five days, rosette forming spheres were transferred into EB2 medium for 28 days. EB2 medium consists of a 1/1 mix of Advanced DMEM/F12 and Neurobasal™ Medium (Invitrogen), supplemented with 1/100 Antibiotic-Antimycotic, 1/200 GlutaMAX™-I Supplement, 1/100 N2 Supplement, 1/200 B-27 Supplement Minus Vitamin A (Invitrogen); 1/100 MEM Non-Essential Amino Acids Solution (10 mM; Invitrogen) and 1.25 μg/ml human Insulin. Organoids were collected for immunohistochemistry, RNA or protein extraction. Table S2 summarizes the batches of organoids used in this study.

To repress SHH signalling during organoid growth, 5 μM Cyclopamine (Cyclopamine, V.californicum, Millipore) was continuously added from the beginning of the EB1 stage (D8) till the end of the organoid culture. 1 μM Purmorphamine (Merk) was used from D8-14 to activate SHH signalling (James et al., 2021).

#### Immunohistochemistry on organoids

For immunohistochemistry, organoids were fixed for 1 hour in 4% paraformaldehyde, incubated in 30% sucrose at +4°C for 24h, embedded in 30% sucrose/OCT mixture (1:1) and frozen on dry ice. Immunofluorescence staining was performed on 10-12 μm cryostat sections as described previously (Theil, 2005) with antibodies against mouse anti-ARL13B (Neuromab 75-287; 1:2000), rabbit anti-ARL13B (Proteintech, 1:200), rabbit anti-COUP-TFII (1:500; provided by M. Studer), rat anti-CTIP2 (1:1000, Abcam #18465), guinea pig anti-DLX2 (1:2000, Bioacademica # 74-116), rabbit anti-EMX1 (1:200; (Briata et al., 1996)), rabbit anti-FOXG1 (1:200; Abcam #18259), guinea pig anti-GLI2 (1:1000; (Cho et al., 2008)), mouse anti glutamylated TUBULIN GT335 (1:1000; AdipoGen, #AG-20B-0020) rabbit anti-GPR161 (1:1000; Proteintech 13398-1-AP), rabbit anti-GSX2 (1:200; Millipore #ABN162), rabbit anti-IFT81 (1:200; Proteintech #11744-1-AP); rabbit anti-IFT88 (1:200; Proteintech #13967-1-AP); rabbit anti-IFT144 (1:200; Proteintech #13647-1-AP); rabbit anti-INPP5E (1:600; Proteintech #17797-1-AP), mouse anti-ISL1/2 (1:100; DSHB clone #39.4D5), mouse anti-NKX2.1 (1:300; Abcam #ab3186), rabbit anti-OLIG2 (1:400; Millipore #AB9610), rabbit anti-PAX6 (1:400, Biolegend #901301), rabbit anti-RPGRIP1L (1:200; Proteintech #55160-1-AP); rabbit anti-SMO (1:600; Proteintech #20787-1-AP), rabbit anti-SOX2 (1:1000; Abcam #92494), rabbit anti-SST (1:200; Peninsula Laboratories # T-4102.0400), rabbit anti-SUFU (1:600; Proteintech #26759-1-AP), rabbit anti-TBR1 (1:400, Abcam #31940), rabbit anti-TBR2 (1:400, Abcam #23345), rabbit anti-TCTN1 (1:200; Proteintech #15004-1-AP); rabbit anti-TMEM67 (1:200; Proteintech #13975-1-AP), mouse anti-γTUB (Sigma T6557; 1:2000), rabbit anti-TULP3 (1:600; Proteintech #13637-1-AP), mouse anti-multi Ubiquitin clone FK2 (MBL International # D058-3).

Primary antibodies for immunohistochemistry were detected with Alexa- or Cy2/3-conjugated fluorescent secondary antibodies. The Tbr1 signals were amplified using biotinylated secondary IgG antibody (swine anti-rabbit IgG) (1:400, BD Biosciences) followed by Alexa Fluor 488 or 568 Streptavidin (1:100, Invitrogen). For counter staining DAPI (1:2000, Life Technologies) was used. Fluorescent and confocal images were taken on a LeicaDM 5500 B fluorescent microscope and Nikon A1R FLIM confocal microscope, respectively.

#### Immunohistochemistry for pluripotency markers

hiPSCs were cultured in Matrigel® Matrix (Corning) coated 24-well plates in Essential 8™ Basal Medium supplemented with Antibiotic-Antimycotic. The cells were grown for an average of 4-6 days until cultures reached around 80% confluence, before they were fixed for 15 min at room temperature in 4% paraformaldehyde/DPBS. Cultures were blocked for 45 min with blocking buffer (BB) containing 6% Goat serum (Dako, S-100) in DPBS, and subsequently incubated with Mouse-anti-Tra-1-60 (1/100, Santa Cruz, sc-21705) antibody diluted in BB for one hour. Cultures were permeabilised with 0.1% Triton-X in DPBS for 10 min, followed by an overnight incubation at 4°C with Rabbit-anti-NANOG (1/800, Cell Signalling, #3580S) and Mouse-anti-OCT3/4 (1/250, Santa Cruz, sc-5279) antibodies diluted in BB supplemented with 0.1% Triton-X. Primary antibodies were detected with Goat-anti-Rabbit-488 (1/1000, Invitrogen, A11008), Goat- anti -Mouse-IgG2b-647 (1/1000, Invitrogen, A21242), Goat- anti -Mouse-IgM-555 (1/1000, Invitrogen, A21426) secondary antibodies. Fluorescent images were captured using a Zeiss observer Z1 microscope.

#### *In situ* hybridization and qRT-PCR

*In situ* hybridisation on 12μm serial cryosections were performed as described previously (Theil, 2005). To generate Digoxigenin-labeled antisense probes, *GLI1* and *PTCH1* cDNAs were PCR amplified using the following oligonucleotides: 5′-TGGACTTTGATTCCCCCACCC-3′ and 5′-ATACATAGCCCCCAGCCCATAC-3′ (*GLI1*); 5′-GGTCTGCCATCCTAACACCC-3′ and 5′-CATGCTAGGTCGCCAATGGT-3′ (*PTCH1*). pBS hSHH (CT#401) was a gift from Cliff Tabin (Addgene plasmid # 13,996) (Marigo et al., 1995). Images were taken on a LeicaDMLB upright compound microscope.

To validate differential expression of *PTCH1* and *GLI1*, total RNA was extracted from control and *INPP5E*^D477N/D477N^ organoids (n=3 samples per genotype) using an RNeasy Plus Micro Kit (Qiagen) and reverse transcribed using Superscript™ IV VILO™ Master ezDNase enzyme (Thermo Fisher Scientific). Quantitative reverse transcription PCR (qRT-PCR) was performed using QuantiFast SYBR Green PCR Kit (Qiagen) and a DNA Engine Opticon System (GRI); the used oligonucleotides are summarized in Table S1. For each sample Ct values were extrapolated using the Opticon software and ratios of relative gene expression levels of *ATP5* (reference gene) and *PTCH1*/*GLI1* were calculated based on a modified ΔΔCt method taking into account different PCR kinetics (Pfaffl, 2001); PCR efficiencies are summarized in Table S1.

#### Western blot

Protein was extracted from control and *INPP5E*^D477N/D477N^ organoids (derived from n=3 control and n=2 mutant lines) as described previously (Magnani et al., 2010). 20 μg protein lysates were subjected to gel electrophoresis on a 3-8% NuPAGE® Tris-Acetate gel (Life Technologies), and protein was transferred to a Immobilon-FL membrane (Millipore), which was incubated with goat anti-h/m GLI3 (1:500, R&D Systems #AF3690) and mouse anti-β-Actin antibody (1:15,000, Abcam #ab6276). After incubating with donkey anti-goat IgG IRDye680RD (1:15,000, LI-COR Biosciences) and donkey anti-mouse IgG IRDye800CW secondary antibodies (1:15,000, Life Technologies), signal was detected using LI-COR’s Odyssey Infrared Imaging System with Odyssey Software. Values for protein signal intensity were obtained using Image Studio Lite Version 4.0. GLI3 repressor and full-length protein levels and the GLI3 repressor/full length ratio were compared between control and mutant organoids using an unpaired t test.

#### Confocal imaging, deconvolution and image analyses

The neuroepithelia of organoids were imaged with a Nikon A1R FLIM confocal microscope with the experimenter blinded to the genotype. Laser power and gain were adjusted to maximise intensity of the staining while avoiding overexposure. The Z-stack contained between 5μm and 15 μm of tissue section imaged in 0.13 μm steps. An optical zoom of x2.26 with pixel size of 0.06 was used to show more detail of the cilia. Deconvolution was performed using Huygenes Essential with the signal to noise ratio adjusted to values between 3 and 40 and the quality threshold set to 0.01.

The fluorescence mean intensity of ciliary markers relative to axonemal ARL13B staining were analysed using ImageJ software. 15 cilia per organoid (3 organoids per genotype) were chosen that had an elongated rather than a stubby shape to prevent the accidental measurement of staining artefacts. For both, ARL13B and the marker of interest, background mean staining intensities were determined and deduced from the respective intensity levels in the cilium. The intensity ratio between the marker of interest and ARL13B was used for statistical analyses, thereby minimising bias that might have originated from a variability in the staining or image acquisition. For statistical analyses, the intensity ratios of all control and mutant organoids were collected in two separate groups.

To quantify the percentage of cilia positive for a ciliary marker of interest, the number of ARL13B positive cilia that were also positive for that marker was determined using the ImageJ Cell Counting plug. 100 cilia each were counted for 3 control and 2 mutant organoids.

#### RNA sequencing

Total RNA was extracted using the QIAGEN RNeasy Plus Micro kit from three control and two mutant lines. After assessing the integrity of the RNA samples with an Agilent 2100 Bioanalyzer, (RIN > 7), all RNAs were further processed for RNA library preparation and sequenced on a NextSeq550 High platform (paired-end, 75 bp reads). FastQC software (http://www.bioinformatics.babraham.ac.uk/projects/fastqc/) was used to check for sequencing quality. Reads were aligned to the human reference genome (genome assembly Homo_sapiens.GRCh38.104 downloaded from www.ensembl.org) and sorted/indexed using STAR alignment (https://www.ncbi.nlm.nih.gov/pubmed/23104886) (Dobin et al., 2013) and Samtools software (http://www.htslib.org/) (Li et al., 2009), respectively. Gene expression quantification was conducted using the featureCounts tool (https://www.ncbi.nlm.nih.gov/pubmed/24227677) (Liao et al., 2014). RStudio (version 1.2.5033) and the DESeq2 package (https://www.ncbi.nlm.nih.gov/pubmed/25516281/, version 1.30.1) (Love et al., 2014) were used for count normalization and differential gene expression analyses. Prinicipal component analyses and hierarchical clustering were applied to normalized count data. One outlier from the control group was removed after inspection of sample cluster plots. Gene annotation was achieved with the biomaRt software package (https://www.ncbi.nlm.nih.gov/pubmed/19617889, version 2.46.3) (Durinck et al., 2009). Differentially expressed genes were selected based on an adjusted p value <0.05 and are summarized in Table S3. RNAseq data have been deposited at EBI: E-MTAB-11437.

### Quantification and statistical analysis

Data were analysed using GraphPadPrism 9 software with n=2-12 organoids for all analyses. Normal distribution was tested with Shapiro-Wilk or D'Agostino-Pearson omnibus normality tests and F-tests were used to test for equal variation. Normally distributed data with equal variance were analysed with unpaired t-tests, but with unpaired t-tests with Welch’s correction if data showed unequal variance. In all other cases, Mann Whitney tests were used. A single asterisk indicates significance of p<0.05, two asterisks indicate significance of p<0.01, three asterisks of p<0.001 and four asterisks of p<0.0001. Graphs show the mean as well as upper and lower 95% confidence intervals. Statistical details can be found in the figure legends; Table S4 provides a detailed summary of descriptive statistics of the tests used.

## Data Availability

mRNAseq raw data have been deposited at ArrayExpress and are publicly available as of the date of publication. The accession number is listed in the key resources table. The paper does not report original code. Any additional information required to reanalyse the data reported in this paper is available from the lead contact upon request.

## Supplemental information

Document S1. Figures S1–S7, Tables S1 and S2, and Data S1

Table S3. Summary of differentially expressed genes, related to Figure 3

Table S4. Summary of statistical analyses, related to Figures 3, 5, 6, S1, S3, S6, and S7

Document S2. Article plus supplemental information
