# Supplementary material for: The ciliary gene INPP5E confers dorsal telencephalic identity to human cortical organoids by negatively regulating Sonic hedgehog signaling
Source: Cell Rep. 2022 May 17;39(7):110811. doi: 10.1016/j.celrep.2022.110811 (PMC9620745; doi:10.1016/j.celrep.2022.110811)
Supplement: Document S1. Figures S1–S7, Tables S1 and S2, and Data S1 [file mmc1.pdf]

Cell Reports, Volume *39*

Supplemental information

The ciliary gene *INPP5E* confers dorsal telencephalic identity to human cortical organoids by negatively regulating Sonic hedgehog signaling

Leah Schembs, Ariane Willems, Kerstin Hasenpusch-Theil, James D. Cooper, Katie Whiting, Karen Burr, Sunniva M.K. Bøstrand, Bhuvaneish T. Selvaraj, Siddharthan Chandran, and Thomas Theil

Cell Reports, Volume *39*

Supplemental information

The ciliary gene *INPP5E* confers dorsal telencephalic identity to human cortical organoids by negatively regulating sonic hedgehog signaling

Leah Schembs, Ariane Willems, Kerstin Hasenpusch-Theil, James D. Cooper, Katie Whiting, Karen Burr, Sunniva M.K. Bøstrand, Bhuvaneish T. Selvaraj, Siddharthan Chandran, and Thomas Theil

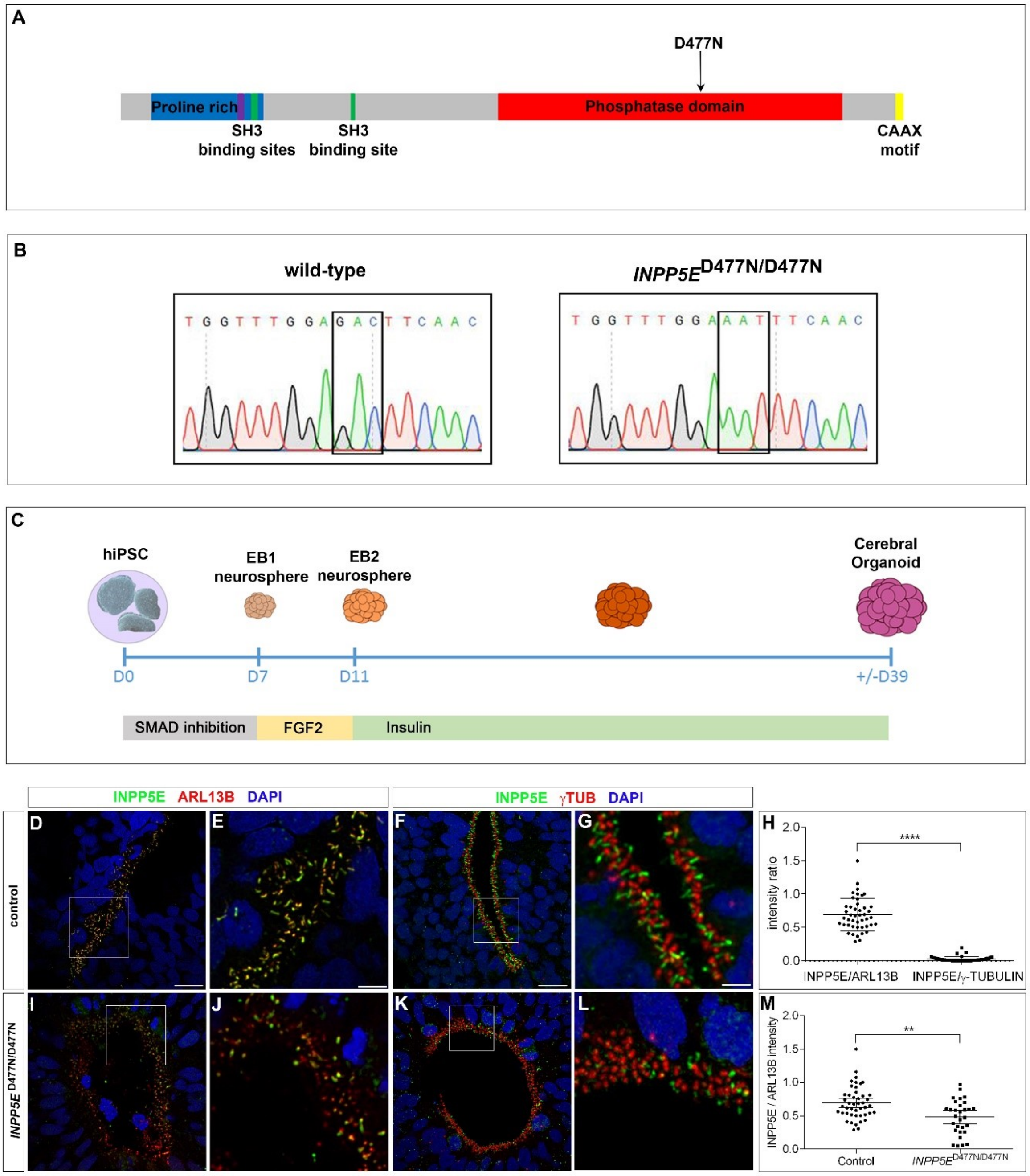

Figure S1: *INPP5E* mutagenesis and expression in cortical organoids. Related to Figure 1 and the STAR methods. (A) Schematic domain structure of the INPP5E protein, the D477N mutation is indicated. (B) Sequencing traces confirming successful mutagenesis. (C) Schematic of the protocol used to generate cortical organoids. (D-M) INPP5E expression in cilia of control (D-G) and in *INPP5E*$^{D477N/D477N}$ organoids (I-L). INPP5E protein was confined to the axoneme and excluded from the basal body with reduced expression levels in *INPP5E*$^{D477N/D477N}$ cilia. (H) Comparison of INPP5E expression in control lines with the axonemal and basal body markers ARL13B and γTUBULIN, respectively. (M) Quantification of the INPP5E/ARL13B fluorescence intensity ratio in control and *INPP5E*$^{D477N/D477N}$ cilia. Statistical data are presented as means ± 95% confidence intervals (CI); Mann-Whitney tests; n = 45 cilia from three different lines (H); n=45 (control) and n=30 (mutant) (M); ** $p < 0.01$; **** $p<0.0001$. Scale bar: 10 μm (E, G), 2.5 μm (D, F).

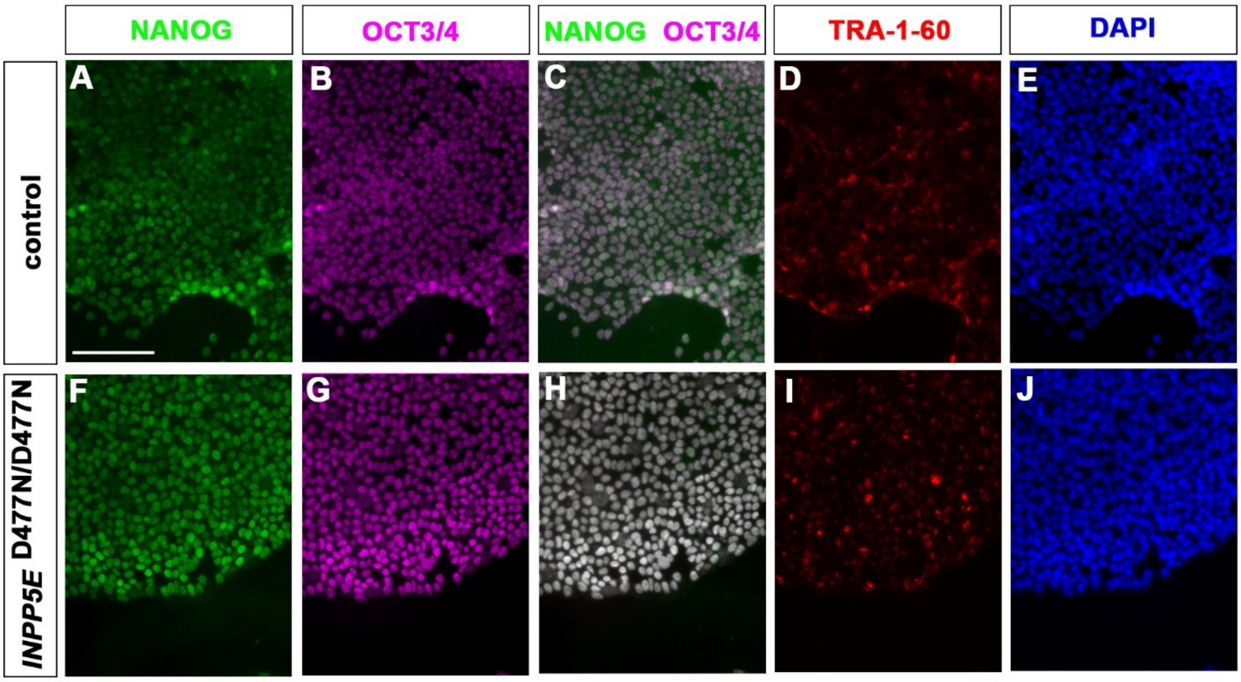

Figure S2: Expression of pluripotency markers in control and *INPP5E*$^{D477N/D477N}$ iPSC lines. Related to the STAR methods. (A-J) Immunofluorescence stainings for the indicated markers. All iPSC lines were positive for NANOG (A, C, F, H), OCT3/4 (B, C, G, H), and TRA-1-60. Scale bar: 100 μm.

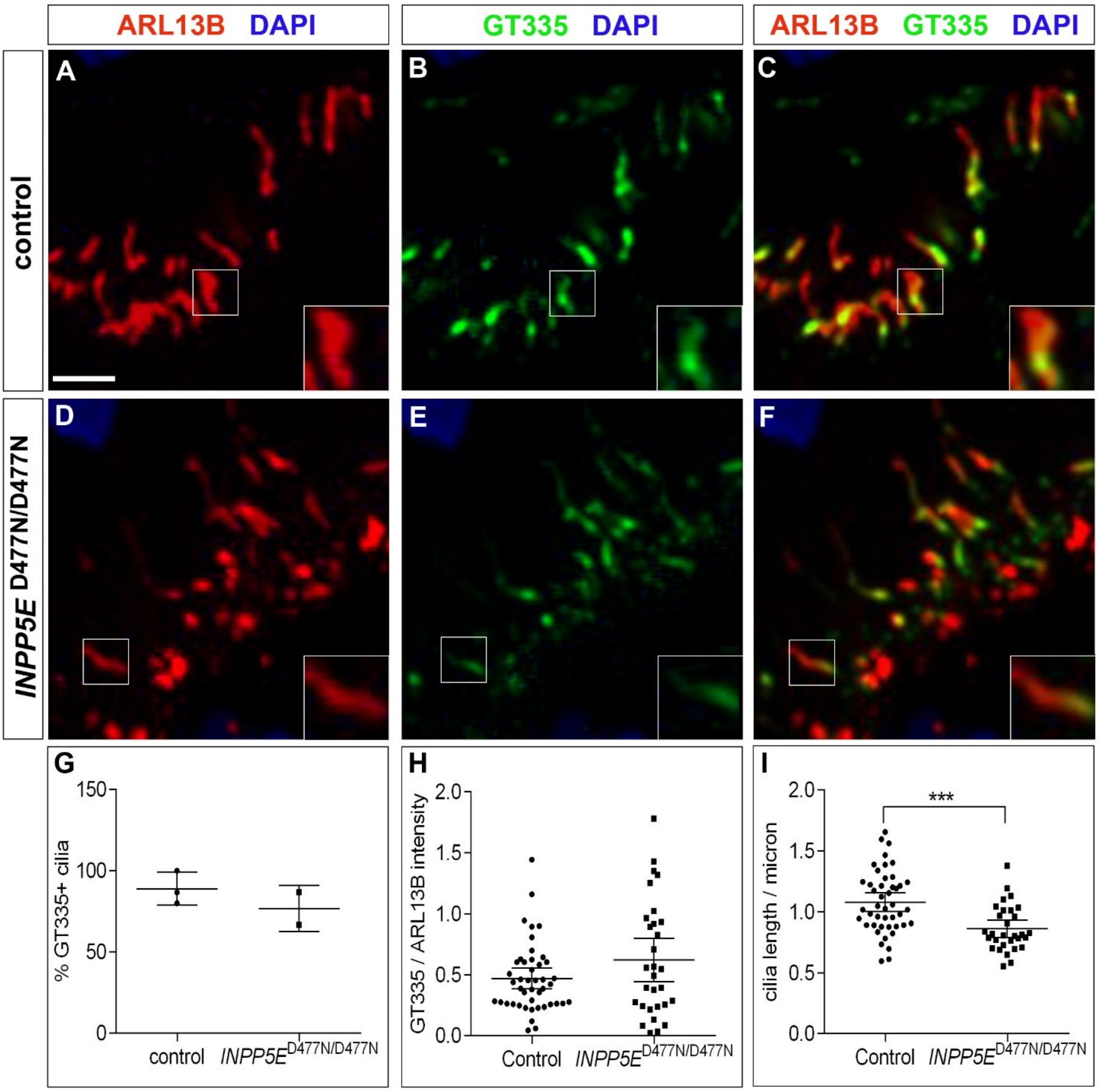

Figure S3: Expression of glutamylated TUBULIN in control and *INPP5E*$^{D477N/D477N}$ organoids. Related to Figure 2. (A-F) Immunofluorescence stainings for the indicated markers. The GT335 antibody detects glutamylated TUBULIN. The inlets show higher magnification pictures of individual cilia. (G-I) Quantification of the frequency of glutamylated TUBULIN positive cilia (G), the glutamylated TUBULIN/ARL13B intensity ratio (H) and of ciliary length (I). Statistical data are presented as means ± 95% confidence intervals (CI); unpaired t-tests (G, I), and Mann Whitney test (H); n=3 (control) and n=2 (mutant) lines for (G); n = 45 (control) and n=30 (mutant) cilia from three and two different lines, respectively (H, I); *** $p < 0.001$;. Scale bar: 2.5 μm.

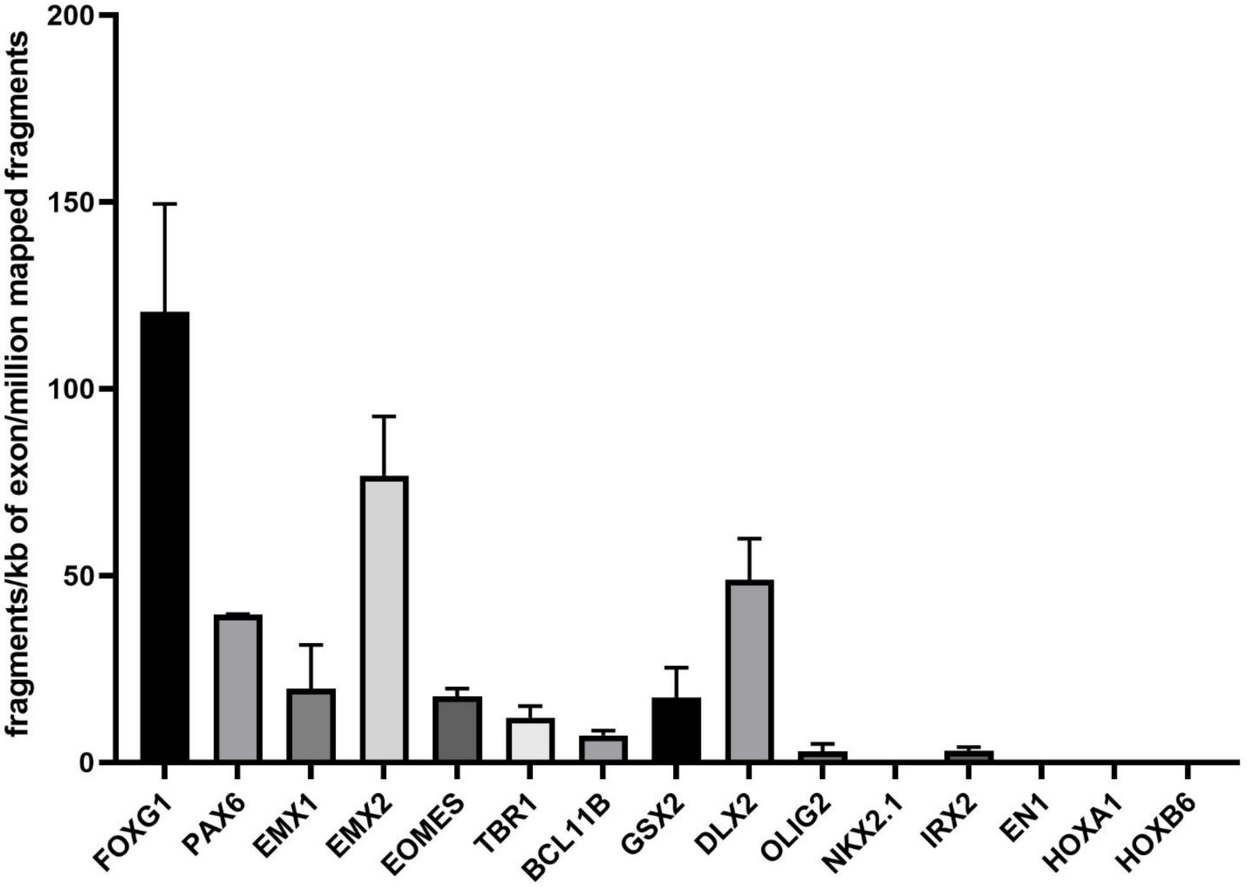

Figure S4: Expression of region specific transcription factors in control organoids. Related to Figure 3. The graph shows the number of read fragments normalized to exon length and to total number of reads for transcription factors characteristic of the telencephalon (FOXG1), dorsal telencephalon (PAX6, EMX1/2, EOMES, TBR1, BCL11B), ventral telencephalon (GSX2, DLX2, NKX2.1), diencephalon (IRX2), midbrain (EN1), hindbrain (HOXA1) and of the spinal cord (HOXB6). Data are presented as means ± SD.

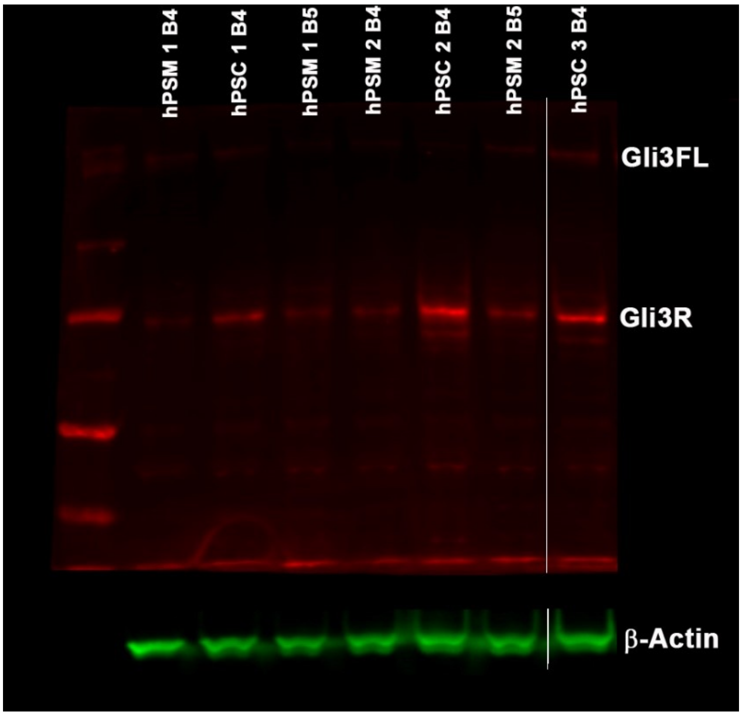

Figure S5: GLI3 Western blot. Related to Figure 3. Organoid tissue was derived from 3 control and 2 mutant lines. B4 and B5 indicate batch number. β-Actin served as a loading control. 2 lanes with protein extract from an additional mutant cell line that was not further analysed were removed from the gel between lane "hPSM2B5" and lane "hPSC3B4".

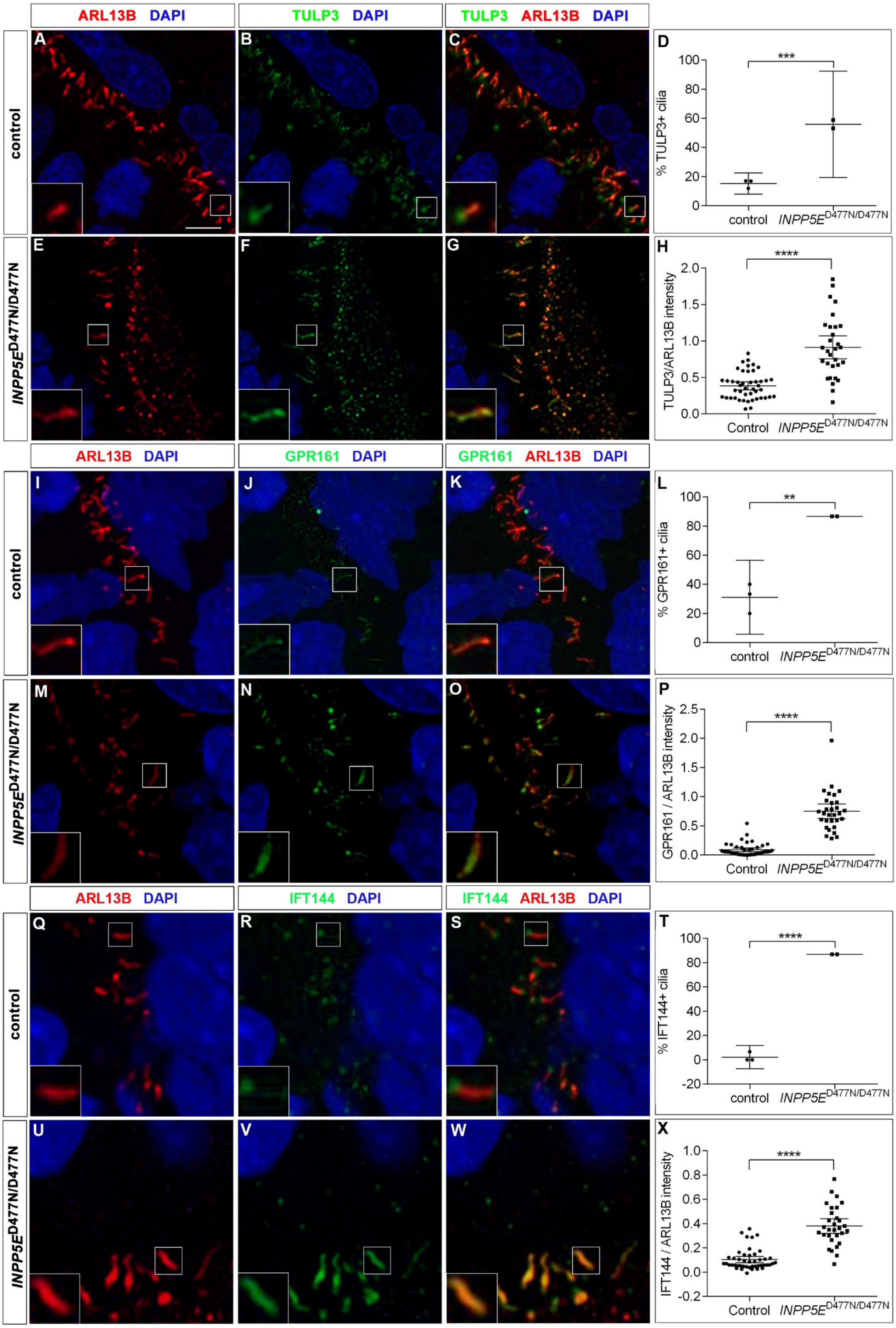
ARL13B DAPI
TULP3 DAPI
TULP3 ARL13B DAPI
control
INPP5E D477N/D477N
% TULP3+ cilia
TULP3/ARL13B intensity
GPR161 DAPI
GPR161 ARL13B DAPI
% GPR161+ cilia
GPR161 / ARL13B intensity
IFT144 DAPI
IFT144 ARL13B DAPI
% IFT144+ cilia
IFT144 / ARL13B intensity
Control

Figure S6: Ciliary localization of TULP3, GPR161 and IFT144. Related to Figures 5 and 6. (A-C, E-G, I-K, M-O, Q-S, U_W) Immunofluorescence analyses of TULP3, GPR161 and IFT144 ciliary expression in control (A-C, I-K and Q-S) and *INPP5E*$^{D477N/D477N}$ organoids (E-G, M-O, U-W). (D, H, L, P, T, X) Quantification of immunostainings. (A-H) TULP3 expression in the axoneme was up-regulated and found in most *INPP5E* mutant cilia but hardly in control cilia. (I-P) GPR161 was expressed at higher levels in almost all cilia in *INPP5E*$^{D477N/D477N}$ organoids. (Q-X) IFT144 accumulated in *INPP5E* mutant cilia. Statistical data are presented as means ± 95% confidence intervals (CI); unpaired t-tests (D, L, T), unpaired t-tests with Welch's correction (H) and Mann Whitney tests (P, X); n=3 (control) and n=2 (mutant) lines for (D, L, T); n = 45 (control) and n=30 (mutant) cilia from three and two different lines, respectively (H, P, X); ** $p<0.01$; *** $p < 0.001$; **** $p<0.0001$. Scale bar: 2.5 μm.

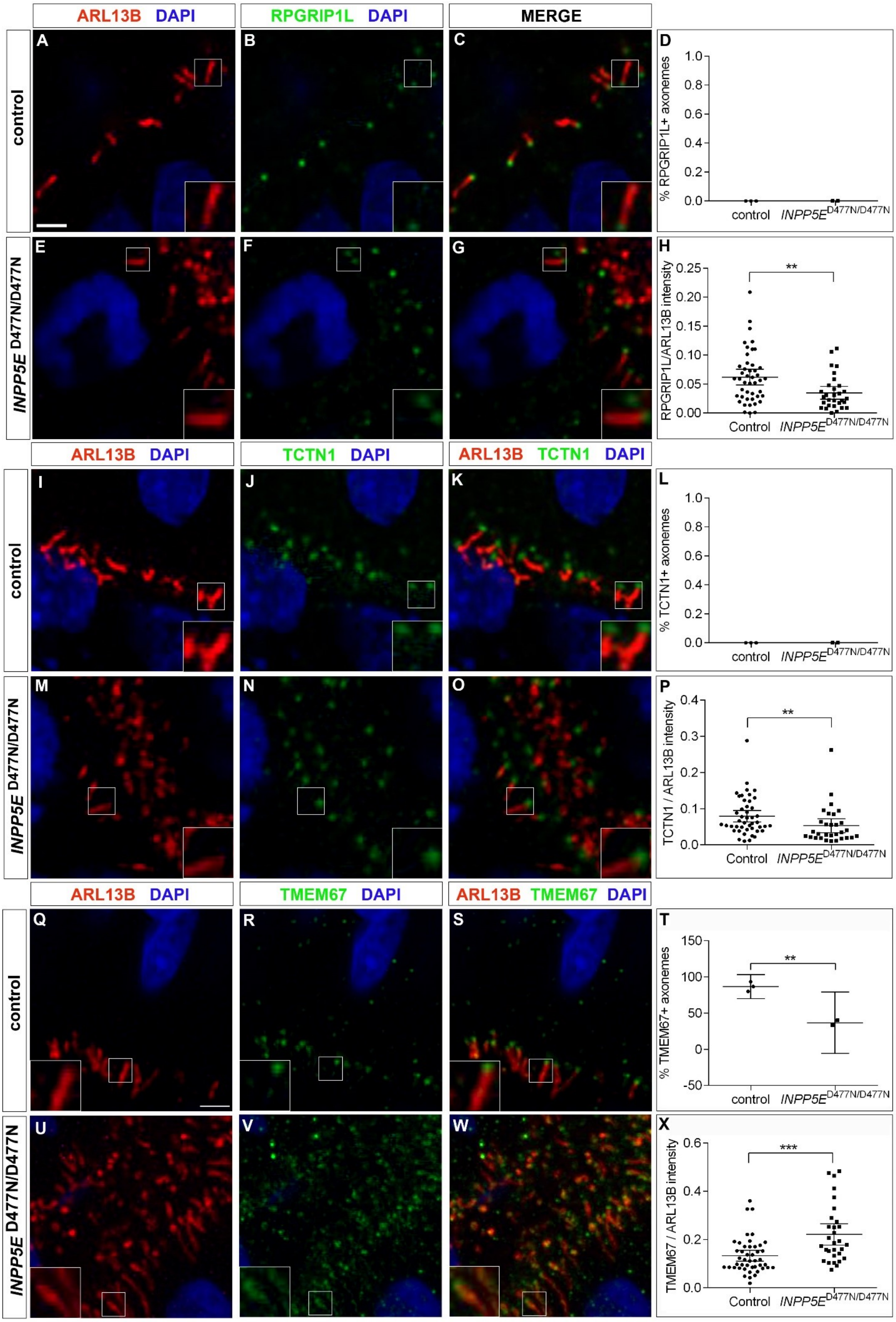

ARL13B DAPI
RPGRIP1L DAPI
MERGE
control
INPP5E D477N/D477N
A
B
C
D
% RPGRIP1L+ axonemes
1.0
0.8
0.6
0.4
0.2
0.0
control
INPP5E D477N/D477N
E
F
G
H
RPGRIP1L/ARL13B intensity
0.25
0.20
0.15
0.10
0.05
0.00
**
Control
INPP5E D477N/D477N
ARL13B DAPI
TCTN1 DAPI
ARL13B TCTN1 DAPI
control
I
J
K
L
% TCTN1+ axonemes
1.0
0.8
0.6
0.4
0.2
0.0
control
INPP5E D477N/D477N
INPP5E D477N/D477N
M
N
O
P
TCTN1 / ARL13B intensity
0.4
0.3
0.2
0.1
0.0
**
Control
INPP5E D477N/D477N
ARL13B DAPI
TMEM67 DAPI
ARL13B TMEM67 DAPI
control
Q
R
S
T
% TMEM67+ axonemes
150
100
50
0
-50
**
control
INPP5E D477N/D477N
INPP5E D477N/D477N
U
V
W
X
TMEM67 / ARL13B intensity
0.6
0.4
0.2
0.0
***
Control
INPP5E D477N/D477N

Figure S7: Transition zone in *INPP5E*$^{D477N/D477N}$ organoids. Related to Figures 5 and 6. (A-C, E-G, I-K, M-O, Q-S, U_W) Immunofluorescence analyses of RPGRIP1L, TCTN1 and TMEM67 ciliary expression in control (A-C, I-K and Q-S) and *INPP5E*$^{D477N/D477N}$ organoids (E-G, M-O, U-W). (A-P) RPGRIP1L (A-H) and TCTN1 (I-P) expression was mainly confined to the transition zone. Low levels of expression in the axoneme were further reduced in mutant cilia (Q-X) The proportion of cilia with TMEM67 expression at the transition zone was decreased while expression of TMEM67 in the ciliary axoneme was increased in mutant organoids.. Statistical data are presented as means ± 95% confidence intervals (CI); unpaired t-test (T), and Mann Whitney tests (H, P, X); n=3 (control) and n=2 (mutant) lines for (D, L, T); n = 45 (control) and n=30 (mutant) cilia from three and two different lines, respectively (H, P, X);; ** $p < 0.01$; *** $p < 0.001$. Scale bar: 2.5 μm.

Data S1: Karyotypic analyses. Related to STAR methods.

ANEUPLOIDY BOBS ASSAY

TDL GENETICS

Genetics report

The Halo Building, 1 Mabledon Place, London WC1H 9AX
T: +44 (0)20 7307 7409 **F:** +44 (0)20 7307 7350
E: tdlgenetics@tdlpathology.com **www.tdlpathology.com**

Patient Details:

Patient's Name : **GS8**
Patient's D.O.B. : **NK**
Dept. Ref. No. : **21G027545**
Patient's Ref. No.: **GS8**
Patient's Gender : **NK**
Clinical Details : **Stem cell analysis**

Referred From: **University of Edinburgh**
Referred By : **Karen Burr**
Sample taken : **NK**
Received : **21/12/21**
Sample Type : **Extracted DNA**

Result:

NO autosomal or sex chromosome aneuploidies were detected in this sample.

Information:

The KaryoLite BoBs (BACs-on-Beads) Kit provided by Perkin Elmer has been used to analyse probes on both the p and q arms of chromosomes 1 to 22, X and Y (q arms in acrocentric chromosomes) to detect whole chromosome aneuploidy.

Triploidies and some mosaics will not be detected by this methodology.

Signed: C. Wl

Registered Clinical Scientist
Dr L J Levett PhD (Director), Dr S Liddle PhD (Head of Genetics)
E Holgado FRCPath, C Lambert

Report Date: **30/12/21**

ANEUPLOIDY
BOBS ASSAY

TDL GENETICS

Genetics report

The Halo Building, 1 Mabledon Place, London WC1H 9AX
T: +44 (0)20 7307 7409 **F:** +44 (0)20 7307 7350
E: tdlgenetics@tdlpathology.com **www.tdlpathology.com**

Patient Details:

Patient's Name	: **CS02 P19**	Referred From:	**University of Edinburgh**
Patient's D.O.B.	: **NK**	Referred By	: **Karen Burr**
Dept. Ref. No.	: **21G024079**	Sample taken	: **NK**
Patient's Ref. No.	: **CS02 P19**	Received	: **09/11/21**
Patient's Gender	: **NK**	Sample Type	: **Extracted DNA**
Clinical Details	: **Stem cell analysis**		

Result:

NO autosomal or sex chromosome aneuploidies were detected in this sample.

Information:

The KaryoLite BoBs (BACs-on-Beads) Kit provided by Perkin Elmer has been used to analyse probes on both the p and q arms of chromosomes 1 to 22, X and Y (q arms in acrocentric chromosomes) to detect whole chromosome aneuploidy.

Triploidies and some mosaics will not be detected by this methodology.

Signed: [signature]
Registered Clinical Scientist
Dr L J Levett PhD (Director), Dr S Liddle PhD (Head of Genetics)
E Holgado FRCPath, C Lambert

Report Date: **11/11/21**

Checked: [initials] Rep time (days): 2

ANEUPLOIDY BOBS ASSAY

TDL GENETICS

Genetics report

The Halo Building, 1 Mabledon Place, London WC1H 9AX
T: +44 (0)20 7307 7409 **F:** +44 (0)20 7307 7350
E: tdlgenetics@tdlpathology.com **www.tdlpathology.com**

Patient Details:

Patient's Name	: **CS25 18N6 P23**	Referred From:	**University of Edinburgh**
Patient's D.O.B.	: **NK**	Referred By	: **Karen Burr**
Dept. Ref. No.	: **21G027197**	Sample taken	: **15/12/21**
Patient's Ref. No.	: **CS25 18N6 P23**	Received	: **16/12/21**
Patient's Gender	: **NK**	Sample Type	: **Extracted DNA**
Clinical Details	: **Stem cell analysis**		

Result:

NO autosomal or sex chromosome aneuploidies were detected in this sample.

Information:

The KaryoLite BoBs (BACs-on-Beads) Kit provided by Perkin Elmer has been used to analyse probes on both the p and q arms of chromosomes 1 to 22, X and Y (q arms in acrocentric chromosomes) to detect whole chromosome aneuploidy.

Triploidies and some mosaics will not be detected by this methodology.

Signed:

Registered Clinical Scientist
Dr L J Levett PhD (Director), Dr S Liddle PhD (Head of Genetics)
E Holgado FRCPath, C Lambert

Report Date: **23/12/21**

ANEUPLOIDY
BOBS ASSAY

TDL GENETICS
Genetics report
The Halo Building, 1 Mabledon Place, London WC1H 9AX
T: +44 (0)20 7307 7409 **F:** +44 (0)20 7307 7350
E: tdlgenetics@tdlpathology.com **www.tdlpathology.com**

Patient Details:

Patient's Name	: **1C2**	Referred From:	**University of Edinburgh**
Patient's D.O.B.	: **NK**	Referred By	: **Karen Burr**
Dept. Ref. No.	: **21G027542**	Sample taken	: **NK**
Patient's Ref. No.	: **1C2**	Received	: **21/12/21**
Patient's Gender	: **NK**	Sample Type	: **Extracted DNA**
Clinical Details	: **Stem cell analysis**		

Result:

NO autosomal or sex chromosome aneuploidies were detected in this sample.

Information:

The KaryoLite BoBs (BACs-on-Beads) Kit provided by Perkin Elmer has been used to analyse probes on both the p and q arms of chromosomes 1 to 22, X and Y (q arms in acrocentric chromosomes) to detect whole chromosome aneuploidy.

Triploidies and some mosaics will not be detected by this methodology.

Signed: C. W

Report Date: **30/12/21**

Registered Clinical Scientist
Dr L J Levett PhD (Director), Dr S Liddle PhD (Head of Genetics)
E Holgado FRCPath, C Lambert

ANEUPLOIDY
BOBS ASSAY

TDL GENETICS

Genetics report

The Halo Building, 1 Mabledon Place, London WC1H 9AX
T: +44 (0)20 7307 7409 **F:** +44 (0)20 7307 7350
E: tdlgenetics@tdlpathology.com **www.tdlpathology.com**

Patient Details:

Patient's Name : **2A6**	Referred From: **University of Edinburgh**
Patient's D.O.B. : **NK**	Referred By : **Karen Burr**
Dept. Ref. No. : **21G027543**	Sample taken : **NK**
Patient's Ref. No.: **2A6**	Received : **21/12/21**
Patient's Gender : **NK**	Sample Type : **Extracted DNA**
Clinical Details : **Stem cell analysis**	

Result:

NO autosomal or sex chromosome aneuploidies were detected in this sample.

Information:

The KaryoLite BoBs (BACs-on-Beads) Kit provided by Perkin Elmer has been used to analyse probes on both the p and q arms of chromosomes 1 to 22, X and Y (q arms in acrocentric chromosomes) to detect whole chromosome aneuploidy.

Triploidies and some mosaics will not be detected by this methodology.

Signed: C. Wll

Report Date: **30/12/21**

Registered Clinical Scientist
Dr L J Levett PhD (Director), Dr S Liddle PhD (Head of Genetics)
E Holgado FRCPath, C Lambert

Table S1: Oligonucleotides used in this study. Related to Figures 3 and S1.

**INPP5E D477N ssODN**	GCCGCAGCGGACGTCACCACCCGCTTCGA TGAGGTGTTCTGGTTTGGAAATTTCAACTTC AGGCTGAGTGGCGGGCGCACAGTCGTGGA ACGCCCTCCTGTGCCAGGGCCTGGTGGTG GACGTGCCGGCGCTGCTGCAGCACGACCA GCTCATCCGGGAGATGCGGAAAGGTG	
**sgRNA**	CTGTGCGCCCGCCACTCAGG	
**INPP5E primers flanking the D477N mutation site**		
Inp D447N Fw	GCGGTTCTTTAGCACGGTTA	
Inp D447N Rev	CTCCTCATCTCCCTCCATG	
**Off-targets**		
chr2-NM_018218_Fw	ATGGAAAACTGAAGGCCTGC	
chr2-NM_018218_Rev	TGCTGTTGTGTCCCTTGTTG	
Chr11-119597300 Fw	AGCTTCCAGGAACCCTTCAA	
Chr11-119597300 Rev	GCACAACCATATCCACCTGC	
Chr11+70329062_Fw	TTGTGTCATAGGTGTGGGCT	
Chr11+70329062_Rev	GGAAGGAGCTGGAAGGGAAT	
Chr16+606525_Fw	TATGAGGCATCGCTGTCAGA	
Chr16+606525_Rev	GGCTGGGATGTAGACTGACA	
Chr1+134123771_NM_052896_Fw	ATGGAAGGTTCTGGAGCACA	
Chr1+134123771_NM_052896_Rev	GTGGTGGAGGCAGTGATAGT	
Chr7+151162603_Fw	ATTGTGCCATTGTGCTCCAG	
Chr7+151162603_Rev	CTCACTGCAGCCTCAAACTC	
**In situ hybridization**		
GLI1 fw	TGGACTTTGATTCCCCCACCC	
GLI1 rev	ATACATAGCCCCCAGCCCATAC	
PTCH1 fw	GGTCTGCCATCCTAACACCC	
PTCH1 rev	CATGCTAGGTCGCCAATGGT	
**qRT-PCR**		**length of PCR product**
hATP5 F2	GTCCAGGGGTATTGCAGGC	112bp
hATP5 R2	TCAGGGATCAGTCCATAACGA	
hGLI1 F2	TACATGTGTGAGCACGAGGG	153bp
hGLI1 R2	TTTTCGCAGCGAGCTAGGAT	

hPTCH1 F2	TGGTTCATCAGAGTGTCGCA	143bp
hPTCH1 R2	GGCATAGGCGAGCATGAGTAA	
**PCR efficiency**		
**Primer combination**		**efficiency**
ATP5	y=-0.29x + 9.66, R^2=0.997	94.98%
PTCH1	y=-0.29x + 10.31, R^2=0.991	94.98%
ATP5	y=-0.30x + 10.12, R^2=0.997	99.53%
GLI1	y=-0.30x + 10.90, R^2=0.991	99.53%

Table S2: Summary of organoid batches used in this study. Related to STAR Methods.

experiment	hPSC1	hPSC2	hPSC3	hPSM1	hPSM2
Figure 2	Batch 1	Batch 3	Batch 3	Batch 1	Batch 1
Figure 3	Batch 1-3	Batch 2, 3	Batch 2, 3	Batch 1-3	Batch 1-3
Figure 4	Batch 1-3	Batch 2, 3	Batch 2, 3	Batch 1-3	Batch 1-3
Figure 5A, B	Batch 4	Batch 4	Batch 4	Batch 4	Batch 4
Figure 5C, D, F, G	Batch 1	Batch 2	Batch 2	Batch 1	Batch 1
Figure 5E, H-L	Batch 4	Batch 4	Batch 4	Batch 4, 5	Batch 4, 5
Figure 6	Batch 2, 3	Batch 2, 3	Batch 2, 3	Batch 2, 3	Batch 2, 3
Figure 7	Batch 1, 2	Batch 2, 3	Batch 2, 3	Batch 1, 2	Batch 1, 2
Figure 8	Batch 1, 2	Batch 2, 3	Batch 2, 3	Batch 1, 2	Batch 1, 2
Figure 9	Batch 1, 2	Batch 2, 3	Batch 2, 3	Batch 1, 2	Batch 1, 2
Figure 10	Batch 1, 2	Batch 2, 3	Batch 2, 3	Batch 1, 2	Batch 1, 2
